# Plasmonic biosensor enabled by resonant quantum tunnelling

**DOI:** 10.1038/s41566-025-01708-y

**Published:** 2025-06-26

**Authors:** Jihye Lee, Yina Wu, Ivan Sinev, Mikhail Masharin, Sotirios Papadopoulos, Eduardo J. C. Dias, Lujun Wang, Ming Lun Tseng, Seunghwan Moon, Jong-Souk Yeo, Lukas Novotny, F. Javier García de Abajo, Hatice Altug

**Affiliations:** 1 Institute of Bioengineering, École Polytechnique Fédérale de Lausanne (EPFL), Lausanne, Switzerland; 2 ICFO-Institut de Ciencies Fotoniques, The Barcelona Institute of Science and Technology, Castelldefels, Spain; 3 Photonics Laboratory, ETH Zürich, Zürich, Switzerland; 4 Institut de Physique et Chimie des Matériaux de Strasbourg, Université de Strasbourg, CNRS, Strasbourg, France; 5 Institute of Electronics, National Yang Ming Chiao Tung University, Hsinchu, Taiwan; 6 School of Integrated Technology, Yonsei University, Incheon, Republic of Korea; 7 BK21 Graduate Program in Intelligent Semiconductor Technology, Yonsei University, Incheon, Republic of Korea; 8 ICREA-Institució Catalana de Recerca i Estudis Avançats, Barcelona, Spain

**Keywords:** Sensors and probes, Metamaterials, Nanophotonics and plasmonics

## Abstract

Metasurfaces provide an ideal platform for optical sensing because they produce strong light-field confinement and enhancement over extended regions that allow us to identify deep-subwavelength layers of organic and inorganic molecules. However, the requirement of using external light sources involves bulky equipment that hinders point-of-care applications. Here we introduce a plasmonic sensor with an embedded source of light provided by quantum tunnel junctions. An optically resonant, doubly periodic nanowire metasurface serves as a top contact for the junction and provides extremely uniform emission over large areas, amplified by plasmonic nanoantenna modes that simultaneously enhance the spectral and refractive index sensitivity. As a proof of concept, we demonstrate spatially resolved refractometric sensing of nanometre-thick polymer and biomolecule coatings. Our results open exciting prospects based on a disruptive platform for integrated electro-optical biosensors.

## Abstract (web summary)

Exploiting resonant quantum electron tunnelling empowered by an optically resonant, doubly periodic plasmonic nanowire metasurface, a biosensor with no external light source is demonstrated, boosting the integrability of the biosensor.

## Main

Plasmonic metal nanostructures have been intensively investigated as a platform for optical sensors owing to their unique abilities to simultaneously support strong optical field enhancement and deep-subwavelength light confinement via localized surface plasmon resonances and propagating surface plasmon polaritons (SPPs)^1^. On the basis of these assets, biosensing devices rapidly excelled the detection performance of conventional optical sensors, paving the way for widespread use and commercialization^2^. Surface plasmon resonance biosensors based on flat metal films have become one of the gold-standard label-free techniques for real-time monitoring of biomolecular interactions in both applied and fundamental bioanalytical studies. Concurrently, nanostructured surfaces and nanoantennas featuring localized plasmonic resonances enabled further enhancement of the sensitivity^3^ and facilitated multiplexed sensing^4^. The compact footprints of such nanoplasmonic biosensors also allowed a reduction in the required sample volumes in portable device configurations^5^ that even enabled the observation of real-time single-cell secretion^6^. More recent advances are exploring quantum plasmonic sensing regimes^7,8^ that uncover new opportunities for enhanced device performance reaching down to the single-molecule detection level^9,10^. Despite the impressive progress experienced by nanophotonics in recent years, the excitation of SPPs mostly requires an external light source combined with bulky coupling schemes, such as prisms, gratings or tightly focusing optics, which limit the usability of plasmonic sensors in biochemical research and medical diagnostics where miniaturized and integrated devices are crucial, especially at point-of-care settings^11^.

Electrical excitation of SPPs constitutes a desirable goal to achieve the ultimate on-chip integration and compact device footprint for applications in biosensing and beyond. In this regard, in 1976, Lambe and McCarthy discovered light generation through electron tunnelling in thin-film heterostructures with a metal (Al)–insulator (Al_2_O_3_)–metal (Au) configuration^12^. These pioneering observations showcased the possibility of direct and ultrafast transduction between electrons and photons, as the excess energy of the tunnelling electrons can generate light via radiative decay assisted by the intermediate excitation of plasmons. Applications of this phenomenon have been mostly associated with scanning tunnelling microscopy operating in ultrahigh-vacuum environments and allowing for the mapping of photon emission with exceptional spatial resolution. In particular, scanning tunnelling microscopy proved itself useful for probing molecular vibrations^13^, visualizing electronic wave functions and molecular orbitals^14,15^, exploring intermolecular coupling^16^ and scrutinizing the dynamics of adsorbed molecules^17^.

More diverse applications of light emission from inelastic electron tunnelling (LIET) are largely hindered by its extremely low intensity, which is the combined result of a low efficiency of the process (on the order of 10^−6^ photons per tunnelled electron) and a small emission area. A large deal of work has been devoted to enhancing the emission intensity from both material science and nanophotonics research viewpoints. One of the critical parameters in devices relying on the LIET process is the quality of the electron tunnel barrier^18,19^. Grain formation and defects of the isolating layer can undermine the stability and efficiency of the insulator junction considerably, thereby necessitating advanced thin-film deposition techniques to form sufficiently smooth layers that are immune to such problems. In this context, two-dimensional (2D) materials offer notable advantages owing to their crystalline structure and atomically flat interfaces. In particular, hexagonal boron nitride with its large band gap (~6 eV) and excellent crystalline quality can serve as a suitable tunnelling barrier material^20,21^, while graphene or transition metal dichalcogenides can act as either electrical contacts^21,22^ or additional modulators of the LIET efficiency, thanks to their optical and electrical characteristics and their tunability via electrical gating^15,23,24^. In addition, barrier engineering through quantum wells has been demonstrated to introduce resonant inelastic electron tunnelling, enhancing the efficiency considerably^25^. However, 2D materials are so far poorly compatible with large-scale fabrication approaches, where a much bigger emission area could compensate for a smaller photon yield.

A major increase in the LIET efficiency can be achieved by using resonant optical nanoantennas. Enhancement of the detected LIET signal from a tunnel junction with a plasmonic nanoantenna was reported experimentally^26^ and later explained theoretically^27^ by showing that the nanoantenna radically increases the electromagnetic local density of states and enhances radiative emission by orders of magnitude, thus overcoming nonradiative decay processes, which are otherwise dominant in conventional tunnel junctions. Additional enhancement of the measured LIET signal is also achieved owing to the ability of nanoantennas to steer the generated radiation towards the detector when they are designed to exhibit a directional emission pattern^26,28,29^. Furthermore, the resonant response of nanoantennas can be tailored such that they shape the spectrum of the inherently broadband LIET emission^26,27,30^.

Assisted by nanophotonic designs and newly available materials, LIET sources have been leveraged for a broad range of applications that include ultra-compact waveguide-integrated light sources^31^, spectroscopic detection of nanoscale distances^32^, on-chip data communication^29^ and enhanced in situ tracking of chemical reactions^33^. Recent studies reported LIET efficiencies surpassing 1% (refs. ^30,34^), achieving uniform emission over a larger area with stable electrical biasing, although minimizing blinking still remains a challenge. Addressing these issues is pivotal for the design of integrated devices incorporating tunnelling junctions in applications such as biosensing.

In this Article, we demonstrate an on-chip self-illuminating label-free optical biosensor that exploits quantum tunnelling in a multilayer metal–insulator–metal film. The top surface incorporates a plasmonic metasurface that plays a dual role, serving simultaneously as an electric contact for the tunnel junction and as an optical interface for facilitating the coupling of inelastic quantum electron tunnelling accompanied by light emission to free-space radiation. The latter aspect, which is enabled by the localized plasmonic modes supported by the antennas in the metasurface, impacts the internal quantum efficiency of the tunnelling process through the enhancement of the radiative component of the electromagnetic density of optical states. This contributes to the improvement of radiative quantum efficiency and, therefore, enhances the detected signal. We make use of a flexible metasurface design optimized for biosensing to produce efficient and spatially uniform LIET, enabling the mapping of the spatial distribution of the analyte layer deposited on the metasurface. These features ultimately enable an integrated nanoscale light source that can transduce small changes of the local optical environment from low volumes of analyte to the modulation of the far-field optical signal without requiring any labels. With plasmonic antennas serving both as a sensing element and a light source, LIET sensor architecture offers a considerably smaller device footprint compared with designs involving the integration of plasmonic structures on top of light-emitting diodes^35^ or photodetectors^36^. Importantly, the inherently low efficiency of LIET is compensated in our design not only by antenna-driven enhancement but also by having a large and uniform lithographically defined area of emission. In our sensing devices, the total emitted power is sufficient for the operation with most general-purpose light detectors. We test our biosensor with various analytes such as thin layers of polymer and biomolecules, and observe that both the intensity and the spectral profile of the emitted light are modulated by the local refractive index changes produced by the presence of the analyte. These results support the use of LIET devices as highly compact and sensitive on-chip optical biosensors for point-of-care applications by eliminating the need for an external light source.

## Results

### Self-illuminating metasurface design

**Fig. 1:**
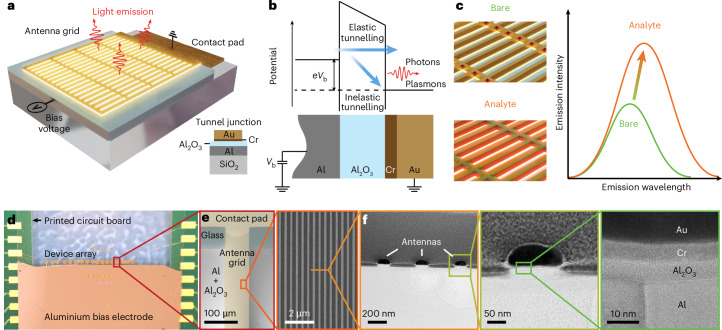
Label-free on-chip biosensor based on light emission from metasurfaces driven by quantum electron tunnelling. **a**, Artistic view of the quantum tunnelling-based sensing device. Inset: the layer sequence; doubly periodic Au metasurface with a thin Cr adhesion layer separated by an Al_2_O_3_ tunnelling barrier from a 25 nm Al film deposited on a glass substrate. **b**, Energy-level diagram for the metal–insulator–metal tunnel junction illustrating the possible electron pathways including plasmon/photon emission driven by inelastic tunnelling. **c**, Illustration of the quantum tunnelling-assisted sensing principle. The bare metasurface (green curve) exhibits a tunnelling luminescence peak that is redshifted and enhanced when the metasurface is covered by an analyte (orange). **d**, Optical microscope image of an array of devices electrically connected to the metallic pads on a printed circuit board. **e**, Enlarged optical image from **d** and SEM image of the metasurface. **f**, High-resolution TEM image of a thin lamella cut from the metasurface. Further zoom-ins of a single antenna and the tunnelling gap allow us to estimate the thicknesses of the Al_2_O_3_ insulator gap (~5 nm) and the Cr adhesion layer (~5 nm).

Our metasurface is based on an interconnected mesh of nanowire nanoantennas that are used as a transducer between the plasmons excited by inelastic electron tunnelling in a vertical planar junction and the far-field light emission (Fig. 1a). In a cross-sectional view of a representative device (Fig. 1a, inset), these cross-linked gold (Au) nanowires reside on top of a thin alumina layer (the tunnelling barrier) that separates it from an aluminium (Al) film (the bottom contact). To make this barrier extremely uniform and ensure a low defect density, we use thermal oxidation of an amorphous Al film evaporated onto a glass substrate, which leads to the formation of a thin Al_2_O_3_ film with self-limited thickness. The metasurface is fabricated by depositing a 50-nm-thick Au film after a thin (~5 nm) chromium adhesion layer. The nanowires have a width of ~92 nm (from scanning electron microscopy (SEM) images), a fixed period of 400 nm along the *x*-axis (horizontal direction), as shown in Fig. 1a, and a varying period (≥100 μm) along the *y*-axis (vertical direction).

A schematic of the resulting tunnel junction is shown in Fig. 1b. In the experiments, a positive bias voltage *V*_b_ is applied to the Au metasurface layer, while the Al layer is grounded. Inelastic electron tunnelling leads to the excitation of plasmons and subsequent emission of photons that are detected in the far field with a spectrometer system for spectral analysis or with a camera for imaging. To electrically drive the sensor, we utilize a custom-printed circuit board with a set of contact pads (Fig. 1d) that are connected to the pads on the chip via wire bonding. The contact pad of each device is fabricated on top of the bare glass area, while the metasurface extends over the bottom Al contact (Fig. 1e). The transmission electron microscopy (TEM) image of a thin lamella cut from the metasurface in Fig. 1f demonstrates a high-quality tunnelling contact with an Al_2_O_3_ barrier thickness of ~5 nm. The proposed design implies no spatial overlap between the analyte on the sensor surface and the emission source (tunnelling gap). While this configuration imposes certain limitations on the sensitivity compared with, for example, picocavity-based sensors^9^, the transduction of optical signal through the more easily accessible plasmonic lattice mode facilitates analyte handling in practice.

### Metasurface electro-optical properties

**Fig. 2:**
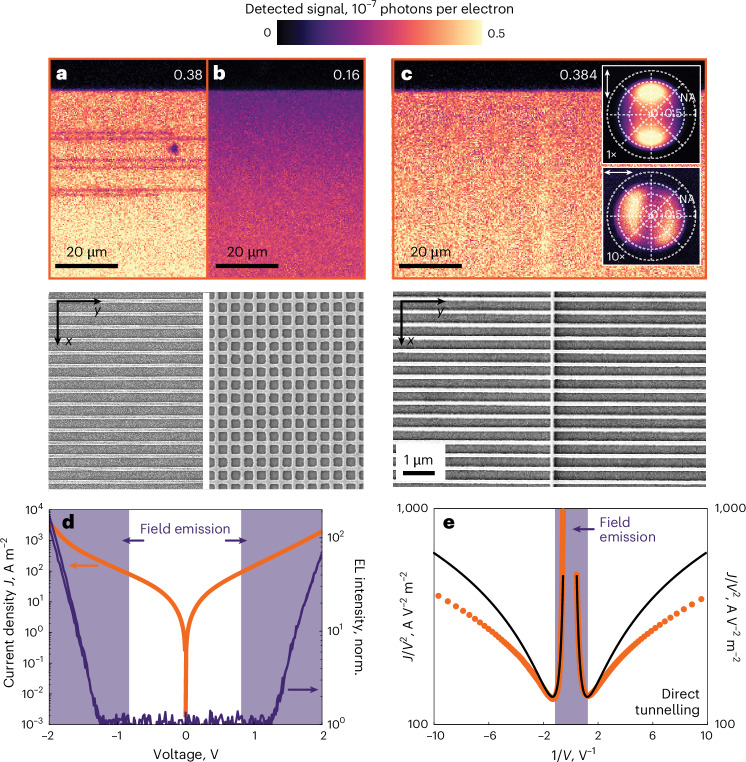
Electro-optical characterization of a large-area metasurface. **a**,**b**, Top: optical microscope images of the light emission from a metasurface consisting of a 1D array of horizontal nanowires with a period of 400 nm in the vertical direction (**a**) and a doubly periodic metasurface formed by densely interconnected 2D arrays of nanowires with a period of 400 nm in both directions (**b**). Bottom: corresponding SEM images. **c**, Top: image of the spatially homogeneous and electrically stable light emission observed from an optimized doubly periodic metasurface formed by less densely interconnected 2D arrays of nanowires with a 400 nm period in the vertical direction and a 100 μm period in the horizontal direction. Insets: measured angular distributions of the tunnelling luminescence with different orientations of the analyser, illustrating that the observed emission has a dipolar nature. Bottom: SEM image of the optimized metasurface. All measurements in **a**–**c** are taken at a voltage of 2.4 V. The numbers in each panel show the average number of photons per electron detected from each structure (in 10^−7^ units). **d**, *I*–*V* curve (orange) of the optimized metasurface in **c** along with the simultaneously recorded electroluminescence (EL) intensity from the tunnelling device (dark violet; normalized (norm.) to the noise level of the detector). The range of voltages above the field emission threshold is shaded in light violet. **e**, Corresponding Fowler–Nordheim representation of the *I*–*V* data (orange curve) and its fitting to the Simmons model (black curve). The field emission region (shaded in violet) is clearly defined by the sharp bend of the curve at 0.8 V. Below 0.8 V (above 1.25 in 1/*V*), the signal is dominated by direct electron tunnelling.

An important prerequisite for sensing applications is to realize a LIET device that produces highly uniform emission over a large detection area for reliable measurements. Owing to the extreme sensitivity of the tunnel junction to local defects, even minor perturbations along the length of the antennas could lead to voltage drops and strong variations in the emission strength. To illustrate this point, we present in Fig. 2a a metasurface made solely from a 1D array of horizontal nanowire antennas with a period of 400 nm. Its LIET image clearly shows such defect-related emission discontinuities. We mitigate this issue by adding an array of nanowires in the *y*-axis direction to form an interconnected mesh (see SEM images in Fig. 2b,c). Overall, we observe that the doubly periodic design suppresses discontinuities, but the density of the interconnections also affects the strength of the emission intensity. For example, the LIET image of a high-density mesh with 2D arrays of nanowire having a period of 400 nm in both vertical and horizontal directions in Fig. 2b produces a very low emission yield. Instead, Fig. 2c shows an optimized metasurface with a period of 400 nm and 100 μm in the *x*- and *y*-axis directions, respectively. For a quantitative comparison of different designs, we estimate the emission efficiency as the total number of detected photons per tunnelling electron. The colour bar in Fig. 2a–c and the average values indicated on top, obtained from a representative sample set at 2.4 V, provide a comparison for three different designs. For the optimized mesh (Fig. 2c), we detected (0.42 ± 0.21) × 10^−7^ photons per electron on average over 20 different tested devices, which is ~2.3 times higher than for the square mesh having a period of 400 nm in both vertical and horizontal directions (Fig. 2b). Also, this metasurface design with less interconnections consistently produced a highly uniform emission over a large area with no emission discontinuities in all tested samples. This is a result of a favourable trade-off between the electrical connectivity that improves with a denser mesh and far-field light outcoupling that is facilitated by sparser antennas. Taking into account the possible decay channels for inelastic electron tunnelling, such as SPP leakage radiation into the substrate and emission at larger angles that are not collected within the numerical aperture (NA) of the objective (see Supplementary Section 2.5 for details), we estimate the total conversion efficiency of our device as 1.2 × 10^−7^ photons per electron, which is consistent with a theoretical estimate in previous literature^37^ (1.1 × 10^−7^). Although this value is lower than the experimentally reported value^37^ (1.6 × 10^−6^), it is compensated by the design of our device providing uniform emission over large areas.

Insets in Fig. 2c present back-focal-plane images of the emission collected from the metasurface for two orthogonal polarizations of the analyser. The radiation patterns show two characteristic lobes that reveal the dipolar nature of the emission associated with the plasmonic mode of the nanowire grid (see Supplementary Section 2.4 for details). The weaker signal with a similar pattern observed for the opposite polarization is consistent with the lower density of nanowire antennas in the orthogonal direction. These observations are supported by calculations of the angle-resolved spectral photonic contribution ${\mathcal{G}}(\omega ,\theta ,\phi )$ (Supplementary Fig. 12).

We further characterize the optimized metasurface through electro-optical measurements. In Fig. 2d, a semilog plot shows the dependence of the current density on the applied bias voltage, exhibiting an exponential profile typical of tunnel junctions^37^. When the applied voltage exceeds 1.25 V, the light intensity detected by a point photodetector increases linearly with the electric current in the shaded violet area. The transition between direct-tunnelling and field-emission regimes is illustrated in the Fowler–Nordheim representation of the *I*(*V*) data in Fig. 2e, which shows a minimum at a voltage of 0.8 V. We fit the *I*–*V* data with the Simmons model^38^, assuming an effective electron mass of 0.23 *m*_e_ (ref. ^39^). From this analysis, we extract the values of the junction’s mean barrier height *φ* = 2.62 eV and barrier width Δ*s* = 3.2 nm. The inconsistency of fitting results with TEM data indicates the presence of local (antenna-scale) inhomogeneities of the barrier layer and surface charge phenomena^40^ that could contribute to diminishing the effective tunnelling barrier width.

**Fig. 3:**
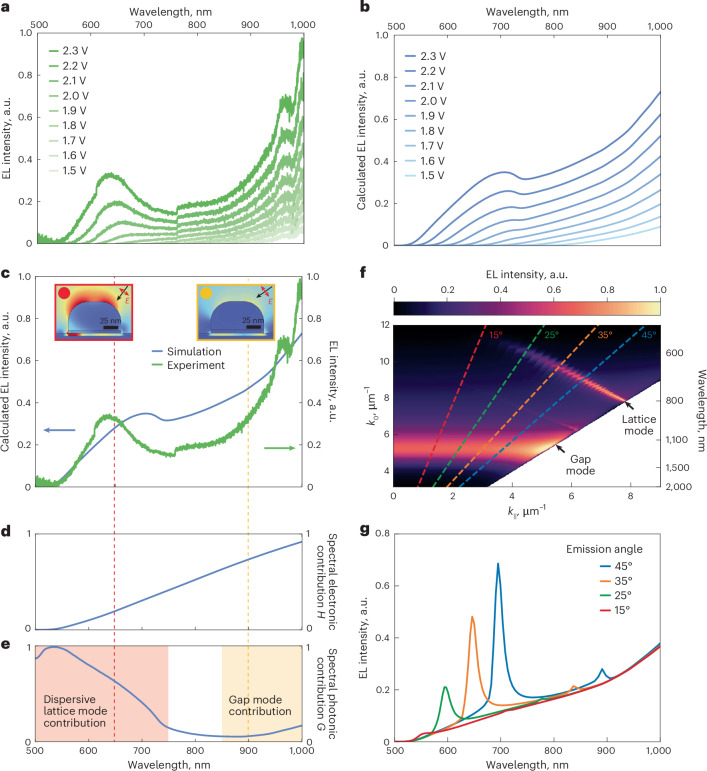
Experimental and theoretical analysis of the spectral response associated with LIET. **a**,**b**, Electroluminescence spectra of the optimized metasurface for various applied voltages ranging from 1.5 V to 2.3 V as obtained from experimental measurements (**a**) and theoretical calculations (**b**). **c**, Calculated spectrum of emission intensity (blue curve) and its correlation to the experimental emission spectrum (green curve) at *V*_b_ = 2.3 V. Insets: the calculated optical electric field amplitude distribution in the *x*–*z* plane for p-polarized plane-wave excitation at two characteristic wavelengths under a certain incident polar angle *θ*: 650 nm (*θ* = 36°; red line) and 900 nm (*θ* = 54°; yellow line). **d**, Calculated normalized spectral electronic contribution *H*(*ω*) for *V*_b_ = 2.3 V. **e**, Calculated normalized spectral photonic contribution *G*(*ω*). **f**, Calculated angle-resolved EL intensity in *k*_0_ − *k*_∣∣_ space. The emission features corresponding to the dispersive plasmonic lattice mode and the dispersionless gap mode are marked with black lines. **g**, Calculated spectra of emission (solid lines) along a discrete set of angles corresponding to the dashed lines of matching colour in **f**.

We then study the optical properties of LIET by measuring the dependence of the emission spectra on the applied bias voltage. Figure 3a shows the corresponding spectra recorded from the optimized metasurface within the 500–950 nm spectral range for a bias voltage *V*_b_ increasing from 1.5 V to 2.3 V. We observe two major trends in the spectra: first, the emission band shifts to shorter wavelengths with increasing *V*_b_, which is explained by the linear change in the cut-off condition with the energy of the emitted photons, *ℏω*_max_ = ∣*eV*_b_∣ (ref. ^37^); second, for high enough *V*_b_ (>1.9 V), the spectra start to manifest a pronounced peak close to 650 nm. The change of *V*_b_ within the 1.5–2.3 V range leads to an increase in the total signal intensity as well as a gradual blueshift of the shorter wavelength peak. At the applied voltage of 2.4 V, the measured emission power density is 0.49 nW mm^−^^2^. This brings the total detected power to 40 pW, making our device feasible for use with most standard detectors.

### Theoretical model

To elaborate on the origin of the observed spectral features and their evolution with bias voltage, we introduce a theoretical model that describes both the tunnelling process and the antenna-assisted coupling of LIET to the optical far field. The spectrally resolved LIET intensity is calculated as the product of two contributions:

1 $${I}_{{\rm{LIET}}}(\omega ,{V}_{{\rm{b}}})\propto H(\omega ,{V}_{{\rm{b}}})\times G(\omega ),$$

where we introduce the spectral electronic contribution *H*(*ω*, *V*_b_) across the tunnelling layer as a function of photon frequency *ω* (that is, the electron energy loss) and applied potential *V*_b_, and the antenna-mediated spectral photonic contribution *G*(*ω*) describing the far-field distribution of photons originating from the Al_2_O_3_ tunnelling layer. Note that *H* depends on the overlap between electronic states in the electron emitter and receptor, as well as the gap distance separating them, but it does not depend on the optical properties of the system. By contrast, *G* encapsulates the optical response, including the far field associated with the transition matrix elements, but it is independent of the electronic matrix elements. Details on the calculations of these two quantities are presented in Methods and Supplementary Information. The results of our simulations closely reproduce the spectral features of the emission observed in the experiment (Fig. 3c) as well as their evolution with increasing *V*_b_ (Fig. 3a,b).

Importantly, the model allows us to discriminate between the relative importance of the spectral electronic contribution *H*(*ω*, *V*_b_) and the antenna-mediated spectral photonic contribution *G*(*ω*) in the resulting spectra. Figure 3d,e shows the spectral dependence of these two functions plotted separately for *V*_b_ = 2.3 V. The spectral electronic contribution *H*(*ω*, *V*_b_) has no pronounced spectral features (Fig. 3d) and its effect on the total emission intensity is a gradual increase towards longer wavelengths. By contrast, *G*(*ω*) (Fig. 3e) exhibits spectral variations associated with the optical modes excited in the structure. The enhancement of *G*(*ω*) in the spectral range from 500 nm to 750 nm originates from the dispersive plasmonic lattice mode. To illustrate this, in Fig. 3f, we plot the calculated angle-resolved emission. The plasmonic lattice mode manifests as an angle-dependent emission peak spanning the range of wave vector *k* between 8 μm^−1^ and 11 μm^−1^. The emission spectra at a discrete set of angles indicated with dashed lines in Fig. 3f are shown in Fig. 3g. The combined contribution of the electronic component *H*(*ω*, *V*_b_) and the photonic component *G*(*ω*) averaged over the angular range corresponding to the NA of the collection objective leads to the formation of the main feature in the total emission (that is, the peak at ~650 nm). This is further detailed in Supplementary Fig. 10 and the related discussion in Supplementary Section 2.3. The angle-resolved spectra also reveal the emission associated with the dispersionless plasmonic gap mode manifested as a peak at around 1,000 nm. Notably, while the Cr layer quenches the contribution from the plasmonic gap mode in the photonic part *G* (Supplementary Figs. 8 and 9), this layer has a positive influence on the total electroluminescence intensity. This is partly due to substantially higher values of the spectral electronic contribution *H* from better matching between the electric band structures of Cr and Al.

Additional simulations of the electric near field of the nanoantenna shown in the insets of Fig. 3c further support the assignment of spectral features of emission to the plasmonic lattice mode and the gap mode. The electric near-field map calculated for p-polarized plane-wave excitation at 650 nm (Fig. 3c, inset in red box) shows two characteristic hotspots at the top surface of the antenna. The minor increase in *G*(*ω*) at longer wavelengths (>850 nm) represents the contribution from the plasmonic gap mode, which has its resonant frequency outside of the spectral range of detection (Supplementary Fig. 11). Accordingly, the near-field distribution for plane-wave excitation at 900 nm (Fig. 3c, inset in yellow box) reveals a maximum field in the Al_2_O_3_ insulating layer.

### Biosensing with a self-illuminating metasurface

**Fig. 4:**
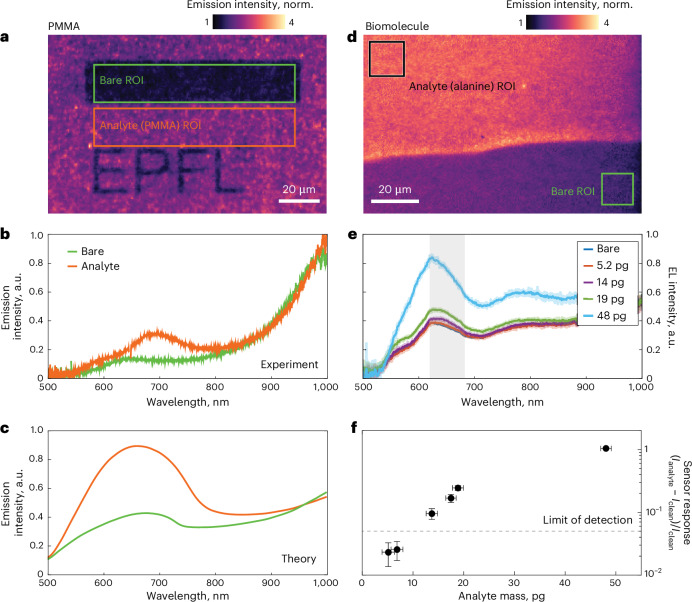
Demonstration of label-free biosensing by LIET in optimized metasurfaces. **a**, Image of the homogeneous light emission from the optimized metasurface with (orange box, analyte region of interest (ROI)) and without (green box, bare ROI) a coating PMMA thin film. **b**, Spectral response from the colour-matched regions in **a**: orange/green with/without PMMA. **c**, Simulated electroluminescence spectra under the conditions of **b**. **d**, Image of light emission from a metasurface partially covered with alanine (black box, analyte ROI) and without alanine (green box, bare ROI). **e**, LIET electroluminescence spectra measured for different amounts of alanine evaporated on the sensor surface. The semitransparent curves show the raw data, while the solid curves show the data smoothed over a window of 2.5 nm (10 points). **f**, Sensor response for different amounts of alanine extracted as a differential emission signal averaged within the 620–700 nm spectral range, highlighted in grey in **e**, with two additional data points at 7 pg and 18 pg. The limit of detection defined as triple the noise level is indicated by a dashed line. The sensor responses are presented as mean values for three samples ± s.d., where s.d. stands for standard deviation from the statistical uncertainty. Alanine thicknesses are presented as mean values ± s.d. derived from AFM measurements.

To benchmark the on-chip LIET-based optical biosensor, we test it with two organic analytes using a polymethyl methacrylate (PMMA) polymer layer and a thin film of amino acid biomolecules. Figure 4a shows the LIET image of the metasurface covered with 45-nm-thick PMMA and biased at 2.8 V. To illustrate the changes in the emission intensity and the spectrum with and without analyte, after spin-coating a uniform layer of PMMA on the sensor surface, we use electron-beam (e-beam) exposure and subsequent development steps to selectively remove the polymer and create PMMA-free regions. Figure 4a shows a rectangularly shaped bare region (green box) that is used for reference spectra measurements as well as a patterned region in the form of the EPFL logo (orange box). The image shows a 2.2-fold enhancement of the emission intensity at ~650 nm on the areas covered with the analyte film compared with the bare regions, and the intensity distribution remains spatially uniform. Further insight is provided by the emission spectra shown in Fig. 4b, which are measured from the bare (green curve) and analyte-covered (orange curve) regions of interest on the functionalized LIET device. The spectra reveal that the stronger emission intensity in the LIET image is mainly due to enhancement of the shorter wavelength peak corresponding to the dipolar plasmon mode of the nanowire antenna.

To elaborate on the origin of the changes in the emission owing to the presence of a PMMA film, we considered a uniform layer with a thickness of 45 nm and a refractive index of 1.49 (ref. ^41^) in the theoretical model used above. The modifications of the spectra observed in the experiment are in good agreement with our simulations based on equation (1) (Fig. 4c). The dominant effect is an increase in the amplitude of the ~650 nm peak, which is well reproduced in the simulations (2.13-fold increase versus 2.2 in the experiment). This intensity change originates from the refractometric spectral redshift of the dispersive plasmonic lattice mode, which improves the coupling of emission into the NA of the objective (see Supplementary Fig. 13 and detailed discussion in Supplementary Section 2.4).

Finally, to demonstrate the biosensing application of our device and quantitatively estimate the sensitivity performance, we studied the dependence of the LIET signal on varying amounts of biomolecules deposited on the surface of the device. For these studies, the antenna width of the sample was slightly changed from 92.5 nm to 120 nm. This new width did not lead to any sizable changes in the spectral profile (Fig. 4b,e), but allowed us to use a 10× (NA = 0.3) objective lens owing to the shift in the dispersion of plasmonic lattice modes towards small angles. In this way, we were able to compare the sensing performance with a state-of-the-art plasmonic biosensor based on Au nanohole arrays that are optimized for low NA optics. As a bioanalyte, we used alanine, which is an α-amino acid with the chemical formula C_3_H_7_NO_2_ and a molecular weight of 89 daltons. Small amounts of alanine were deposited onto the sample surface through thermal evaporation. During the evaporation process, we used a shadow mask to block a portion of the structure, creating an uncovered region with a well-defined boundary that served as an in-line reference (see Supplementary Section 1.2 for details). Figure 4d shows a LIET image of the sensor surface partially covered with alanine. Similar to the PMMA data shown in Fig. 4a, the analyte-covered region exhibits an increase in the LIET intensity. To test the limit of detection of our sensor, we performed measurements for different amounts of biomolecules ranging from ~5 pg to ~50 pg (see Supplementary Section 1.2 for an estimation of the analyte amount). Figure 4e shows the response of the sensor to different doses of evaporated analyte, indicating a gradual increase in the detected emission intensity for higher amounts of alanine. As an output metric for our sensor, we used the LIET intensity. This holds several advantages over spectral shift characterization, namely simpler detection principle, higher signal-to-noise ratio and faster response time. We integrated the emission within a spectral region from 620 nm to 700 nm because this yielded the strongest modulation in the LIET signal, which in practice can be realized by introducing a spectral band-pass filter in the detection scheme. The extracted data are plotted in Fig. 4f, normalized to the integrated emission of the bare chip. Our measurements indicate a reliable detection of analyte down to ~9 pg within the signal collection area, defined as a mass for which the response of the sensor is 3 times larger than the noise level. To correlate the performance of our device with state-of-the-art methods, we used an optimized nanoplasmonic biosensor consisting of Au nanohole arrays with 630 nm RIU^−1^ of bulk sensitivity^42^, which is well within the range reported in the literature^43^ (see Supplementary Section 1.2 for details). The results show a similar signal amplitude, highlighting the potential of our LIET platform for high-performance biosensing.

## Discussion

In brief, we introduce a self-illuminating plasmonic sensor in which light emission is provided by quantum electron tunnelling junctions. Our design features a bottom Al electrode, with a thin isolating layer of alumina formed by thermal oxidation of the film and acting as a tunnelling barrier. The upper electrode consists of a doubly periodic metasurface made of resonant Au nanowire antennas that simultaneously provide enhanced electron-to-light conversion and far-field light emission owing to the mediation of plasmonic antenna modes. We optimize the metasurface to provide exquisite spatial uniformity and large-area emission, which are critical for a good performance of the proposed sensors.

The electro-optical properties of the devices and their spectral performance towards the detection of different types of analyte are well explained by theoretical modelling incorporating microscopic details of the tunnelling process as well as the electromagnetic response associated with plasmonic antenna modes. We demonstrate that the emission peak at short wavelengths, which originates from the resonant mode of the nanowire antennas, allows spatially resolved refractometric sensing with our device. We validate this sensing concept for a thin film of polymer and a biomolecule layer, for which we demonstrate a limit of detection of ~9 pg.

Beyond the present results, we envision that additional insight could be gained by resorting to angle-resolved photodetection, as the angular distribution of the emission is intimately related to the frequency of the excited modes. Indeed, the evolution of the observed angle-resolved emission patterns (Supplementary Fig. 5) indicates the possibility of photodetection along selected angular windows as a way to gain spectral selectivity without the need to use an optical spectrometer. In addition, because the used metal films are polycrystalline, the effect of conservation of in-plane electron momentum in the tunnelling process is substantially erased by averaging over grain orientations (Supplementary Fig. 6). We thus anticipate that even more control over the inelastic tunnelling process could be attained with the use of crystalline materials^23^ by exploiting the in-plane momentum-matching condition of the tunnelling electrons defined by the mutual orientation of the crystallographic axes in the two metals. Finally, we note that the planar geometry of our device is compatible with wafer-scale fabrication, while the low efficiency of inelastic tunnelling is compensated by antenna-driven enhancement and a large area of emission. This opens up exciting prospects to realize a practical electro-optical biosensor and novel device applications.

## Methods

### Fabrication of the sensor

The LIET device was fabricated on a commercially available glass coverslip measuring 22 × 22 × 0.13 mm^3^. The bottom metal electrode was designed using ultraviolet laser photolithography (VPG 200, Heidelberg Instruments), while the metasurface and top electrode were defined by using e-beam lithography (Raith EBPG5000+). Each metal thin film was deposited through e-beam evaporation using a Leybold Optics LAB 600H evaporator. The bottom electrode was formed by depositing Al through the designed photoresist layer and subsequently lifting it off. Then, the Al_2_O_3_ layer was formed using a thermal oxidation process in a Neytech furnace (Qex) at 200 °C for 3 h. After forming the Al_2_O_3_ tunnel barrier, we deposited a Cr adhesion layer followed by deposition of the top Au electrode using a patterned e-beam resist. Subsequently, the e-beam resist layers were removed through a lift-off process, resulting in the formation of a metasurface connected with a top electrode. We used 92 nm wide antennas for electro-optical characterizations and 120 nm wide antennas for sensing experiments with alanine. Electrical contacts from the bottom and top Al and Au electrodes were established by connecting them to a printed circuit board with Au wire using a wire bonder (F*&*S Bondtec 5630i Semiconductor GmbH). All sample fabrication and preparation procedures took place within the cleanroom facilities at the Center of MicroNanoTechnology (CMi) at EPFL.

### Preparation of polymer and biomolecular analyte layers on LIET devices

For the on-chip LIET-based optical biosensor, we prepared two types of analyte: PMMA and thin layer of amino acid molecules (alanine). For the former, we used PMMA 495k A2 (Sigma-Aldrich), which was coated onto the substrate using a spin-coating machine at 6,000 rpm for 60 s. Subsequently, the sample was baked at 180 °C for 5 min. To open an area for reference measurements, the PMMA layer was patterned using an e-beam. After the exposure, the resist was developed using a MiBK:IPA = 1:3 solution for 1 min. The resulting analyte layer had a thickness of approximately 45 nm and a refractive index of 1.49.

For deposition of alanine (Thermo Scientific), we used thermal evaporation. Approximately 100 mg of alanine was loaded into the evaporation boat for each process. The sample, mounted on a dummy wafer, was positioned approximately 40 cm above the evaporation boat containing the alanine. The evaporator chamber was evacuated to a pressure of 10^−5^ mbar. To initiate evaporation, an electrical current was applied to heat the evaporator boat. The evaporation rate and the amount of deposited material were monitored using a quartz crystal microbalance. To ensure a uniform coverage on the sample, the evaporation rate was maintained between 0.1 Å s^−1^ and 0.3 Å s^−1^. The resulting alanine layer was characterized using atomic force microscopy (AFM) on a polished reference silicon chip placed near the studied sample during the same deposition session.

### Sample characterization

Optical measurements were conducted using a customized inverted Nikon microscope (Ti-E). For studies of the electro-optical response of our devices, we used a 50× NA 0.8 objective, except for the sensing experiments, where we used a 10× NA 0.3 objective. The spectrally resolved measurements were performed with an IsoPlane 320 spectrometer using a Pixis camera from Princeton Instruments. All spectra were normalized to account for system efficiency, acquisition time and sampling rate, resulting in wavelength-resolved emission spectra quantified in units of counts s^−1^ nm^−1^. Real-plane and back-focal-plane images of the metasurface emission were captured using an Andor iXon Ultra EMCCD camera (model 888). For the experiments, we customized the optical path in the signal collection channel by introducing an additional linear polarizer and a Bertrand lens before the tube lens that formed the image on the EMCCD camera. Switching between real-plane and back-focal-plane imaging regimes was achieved by adding or removing the Bertrand lens from the optical path.

Electrical biasing was performed using a Keithley 2636B sourcemeter along with Kickstart 2 software from Linktronix. Material characterization was conducted through various techniques, including SEM (MERLIN Zeiss Gemini II), TEM (Tecnai Osiris) and AFM (Bruker FastScan).

### Theoretical calculations

The LIET photon emission intensity is modelled as

$${I}_{{\rm{LIET}}}(\omega ,{V}_{{\rm{b}}})\propto H(\omega ,{V}_{{\rm{b}}})\times G(\omega )$$

(equation (1) in the main text), which is the product of two independent quantities: an electronic contribution *H*(*ω*, *V*_b_) and the antenna-mediated photonic contribution *G*(*ω*). The former is related to the electron tunnelling probability under the bias voltage *V*_b_ and it effectively accounts for the generation of a tunnelling current, while the latter describes the intensity of far-field radiation originating from the tunnelling layer owing to inelastic electron tunnelling.

When a positive bias voltage *V*_b_ is applied to the Au/Cr ribbon and the Al substrate layer is grounded, electrons tunnel from the Al layer to the Cr layer. Therefore, the tunnelling probability depends on (i) the available electronic states in both Al and Cr, given by the respective density of states ${\rho }_{E}^{{\rm{Al}}}$ and ${\rho }_{E}^{{\rm{Cr}}}$ as a function of electron energy *E*, and (ii) their occupation fractions, determined by the Fermi–Dirac distributions ${f}_{E}^{\;{\rm{Al}}}$ and ${f}_{E}^{\;{\rm{Cr}}}$ written as ${f}_{E}={[{e}^{(E-\mu )/{k}_{{\rm{B}}}T}+1]}^{-1}$, where *μ* is the chemical potential for each of the metals (Ag and Cr), *k*_B_ is the Boltzmann constant and *T* = 300 K is the electron temperature (see more details on these quantities in Supplementary Fig. 15). The electronic contribution *H*(*ω*, *V*_b_) is then proportional to

2 $$H(\omega ,{V}_{{\rm{b}}})\propto \int\,d{E}_{{\rm{f}}}\int\,d{E}_{{\rm{i}}}\,{\rho }_{{E}_{{\rm{i}}}}^{{\rm{Al}}}\,{\rho }_{{E}_{{\rm{f}}}}^{{\rm{Cr}}}\,{f}_{{E}_{{\rm{i}}}}^{\;{\rm{Al}}}\,\left(1-{f}_{{E}_{{\rm{f}}}}^{\;{\rm{Cr}}}\right)\,\delta ({E}_{{\rm{i}}}-({E}_{{\rm{f}}}+\hslash \omega )+e{V}_{{\rm{b}}}),$$

where *e* is the elementary charge, the integrals run over all possible initial (*E*_i_) and final (*E*_f_) electron energies, and the Dirac *δ*-function enforces energy conservation. The variation of *H*(*ω*, *V*_b_) with both light wavelength (*λ* = 2*πc*/*ω*) and bias voltage is shown in Supplementary Fig. 6, exhibiting a gradual increase towards longer wavelengths and for higher bias voltages. We note that, in addition to energy conservation, parallel momentum conservation of the tunnelling electrons with respect to the metallic surface must be equally imposed, but the result is only dependent on the electronic densities of states when averaging over crystal surface orientations (see comparison between calculations with and without inclusion of in-plane wave-vector conservation in Supplementary Fig. 6). Importantly, electron tunnelling is modelled between Al and Cr. We have also performed calculations to estimate the influence of Au on this process as shown in Supplementary Section 2.2. On the basis of these results, we conclude that the approximation of neglecting the influence of Au in the evaluation of the tunnelling matrix elements is rather accurate.

The antenna-mediated photonic contribution *G*(*ω*) describes how the transition dipoles generated in the tunnelling layer are able to radiate into the far field. This quantity can be written as $G(\omega )\propto \int\,d\theta \,\sin \theta \int\,d\phi \,{\mathcal{G}}(\omega ,\theta ,\phi )$, where we introduce

3 $${\mathcal{G}}(\omega ,\theta ,\phi )=\sum _{l}{\left\vert \sum _{j}g(\hat{{\bf{r}}},{{\bf{r}}}_{lj},\omega )\right\vert }^{2}$$

as the angle-resolved photonic contribution. Here $g(\hat{{\bf{r}}},{{\bf{r}}}_{lj},\omega )$ represents the far-field amplitude generated in a direction $\hat{{\bf{r}}}$ (pointing along the polar and azimuthal angles *θ* and *ϕ*) by a unit dipole placed at **r**_*lj*_ within the tunnelling Al_2_O_3_ layer under the antenna region, where *l* and *j* are indices indicating the dipole position along the *x* and *y* directions, respectively (that is, we define $g(\hat{{\bf{r}}},{{\bf{r}}}_{lj},\omega )$ such that the far field generated along a direction $\hat{{\bf{r}}}$ by a unit dipole oscillating with frequency *ω* at position **r**_*lj*_ is written as *e*^*iωr*/*c*^*g*/*r*, where *r* is the distance from the sample to the detector). Incidentally, the reciprocity theorem ensures that $g(\hat{{\bf{r}}},{{\bf{r}}}_{lj},\omega )$ can be obtained from the field generated at the position **r**_*lj*_ by a light plane wave impinging at the structure from a direction $-\hat{{\bf{r}}}$ and with the same polarization as the emitted far field. Taking advantage of this symmetry, we calculate $g(\hat{{\bf{r}}},{{\bf{r}}}_{lj},\omega )$ from the near field induced at the position **r**_*lj*_ under p-polarized plane-wave illumination from an incidence direction $-\hat{{\bf{r}}}$ at frequency *ω*. We note that the generated dipoles owing to the tunnelling process point perpendicularly to the antenna surface, and therefore, the field emitted by them is p-polarized. We use an electromagnetic numerical solver (COMSOL Multiphysics) to obtain the near field. The quantities ${\mathcal{G}}(\omega ,\theta ,\phi )$ (plotted in Supplementary Fig. 12a for different values of *ω*) and *G*(*ω*) (plotted in Fig. 3e) are calculated from the equations above. To connect with the experimental results, the polar angle *θ* is restricted to a maximum angle ${\theta }_{\max }={\sin }^{-1}({\rm{NA}})$ imposed by the numerical aperture NA = 0.8 and NA = 0.3 of the objectives used in the experiments for electro-optical characterizations and sensing, respectively. For the sake of completeness, we have included momentum-space absorption maps calculated via the Fourier modal method, shown in Supplementary Fig. 12b. These maps are in excellent agreement with the results obtained using the finite-element method (COMSOL Multiphysics) and a Fourier modal analysis.

## Online content

Any methods, additional references, Nature Portfolio reporting summaries, source data, extended data, supplementary information, acknowledgements, peer review information; details of author contributions and competing interests; and statements of data and code availability are available at 10.1038/s41566-025-01708-y.

## Supplementary information

Supplementary Information Supplementary Figs. 1–15 and discussion on device fabrication, materials and characterization (Sections 1.1–1.4) and electroluminescence modelling (Sections 2.1–2.6).

## Data Availability

The data supporting the findings of this study are available within the article and its Supplementary Information files as well as from the corresponding author on reasonable request.
