# Supplementary material for: Plasmonic biosensor enabled by resonant quantum tunnelling
Source: Nat Photonics. 2025 Jun 26;19(9):938–45. doi: 10.1038/s41566-025-01708-y (PMC12411226; doi:10.1038/s41566-025-01708-y)
Supplement: Supplementary file 1 — Supplementary Figs. 1–15 and discussion on device fabrication, materials and characterization (Sections 1.1–1.4) and electroluminescence modelling (Sections 2.1–2.6). [file 41566_2025_1708_MOESM1_ESM.pdf]

nature photonics

Supplementary information https://doi.org/10.1038/s41566-025-01708-y

Plasmonic biosensor enabled by resonant quantum tunnelling

In the format provided by the authors and unedited

Contents

S1 Device fabrication, materials, and characterization **S1**

S1.1 Fabrication process S1

S1.2 Analyte characterization S2

S1.3 Electroluminescence characterization S6

S1.4 Stability of electrical and optical properties S8

S2 Electroluminescence modeling **S9**

S2.1 In-plane momentum conservation S9

S2.2 Calculation of the tunneling matrix elements S10

S2.3 Optical modes supported by the system S13

S2.4 Electromagnetic mode analysis through the angle-resolved emission S17

S2.5 Contribution of the leakage radiation channel S20

S2.6 Electronic band structure and density of states S21

References **S22**

S1 Device fabrication, materials, and characterization

S1.1 Fabrication process

The LIET device was fabricated on a commercially available glass coverslip measuring $22 \times 22 \times 0.13$ mm^3, as illustrated in the process flow in Fig. S1a-g. Before delineating the electrode with nanostructures, a bulk alignment marker was defined using UV laser photolithography (VPG 200, Heidelberg Instruments, Germany) with a Bi-layer resist of LOR 5A / AZ1512HS (Kayaku advanced materials, MicroChemicals), as shown in Fig. S1a. Following this process, an adhesion layer of 5 nm Cr layer and a thin film of 100 nm Au (colored violet) were deposited, and the photoresist was developed using MIF 712. After creating an alignment

marker, the bottom electrode was defined using the same lithography tool and photoresist, as shown in Fig. S1b. Subsequently, a 25 nm layer of Al was deposited via an e-beam evaporator (base pressure of 1.8×10^{-6} mbar) at a deposition rate of 4.0 Å/s (Leybold Optics LAB 600H), followed by liftoff of the resist. Next, the Al_2O_3 layer (acting as the electron tunneling barrier in our devices) was formed through thermal oxidation in a Neytech furnace (Qex) at 200°C for 3 hours, with a ramping rate of 50°C/min (see Fig. S1c). The sample was extracted from the furnace upon reaching room temperature. The stability of the tunnel junction was notably sensitive to this final temperature, and therefore, we adhere to this protocol to ensure stability of the tunnel junction. After defining the Al_2O_3 tunnel barrier, e-beam lithography (Raith EBPG5000+) was employed to delineate a nanomesh structure with a top electrode. The e-beam resist for this pattern consisted of a double layer using MMA EL6 and PMMA 950 K A2. To prevent the e-beam charging effect on these two resists, a thin Au layer was sputtered on top of the e-beam resist, as shown in Fig. S1d. Following e-beam writing and development of these two layers, as shown in Fig. S1e, the adhesion layer of 5 nm Cr and the 50 nm Au layer were deposited using an e-beam evaporator, and the resist was lifted off using a suitable solution (MiBK:IPA=1:3 solution), as shown in Fig. S1f. The final device was formed as shown in Figs. S1g and S1f (cross-sectional and top views, respectively). All sample fabrication and preparation procedures took place within the cleanroom facilities at the Center of MicroNanoTechnology (CMi) in EPFL. The geometrical parameters of the device were analyzed using a bright-field high-resolution transmission electron microscope (HRTEM, Fig. S1h), and then, the composition of each layer was measured by energy dispersive spectroscopy (EDS) in scanning transmission electron microscopy (STEM) mode (Fig. S1j).

S1.2 Analyte characterization

We conducted an investigation into the functionalization of two analytes, PMMA and bioanalyte, on the LIET device using an optical microscope, an EMCCD-based real-plane image,

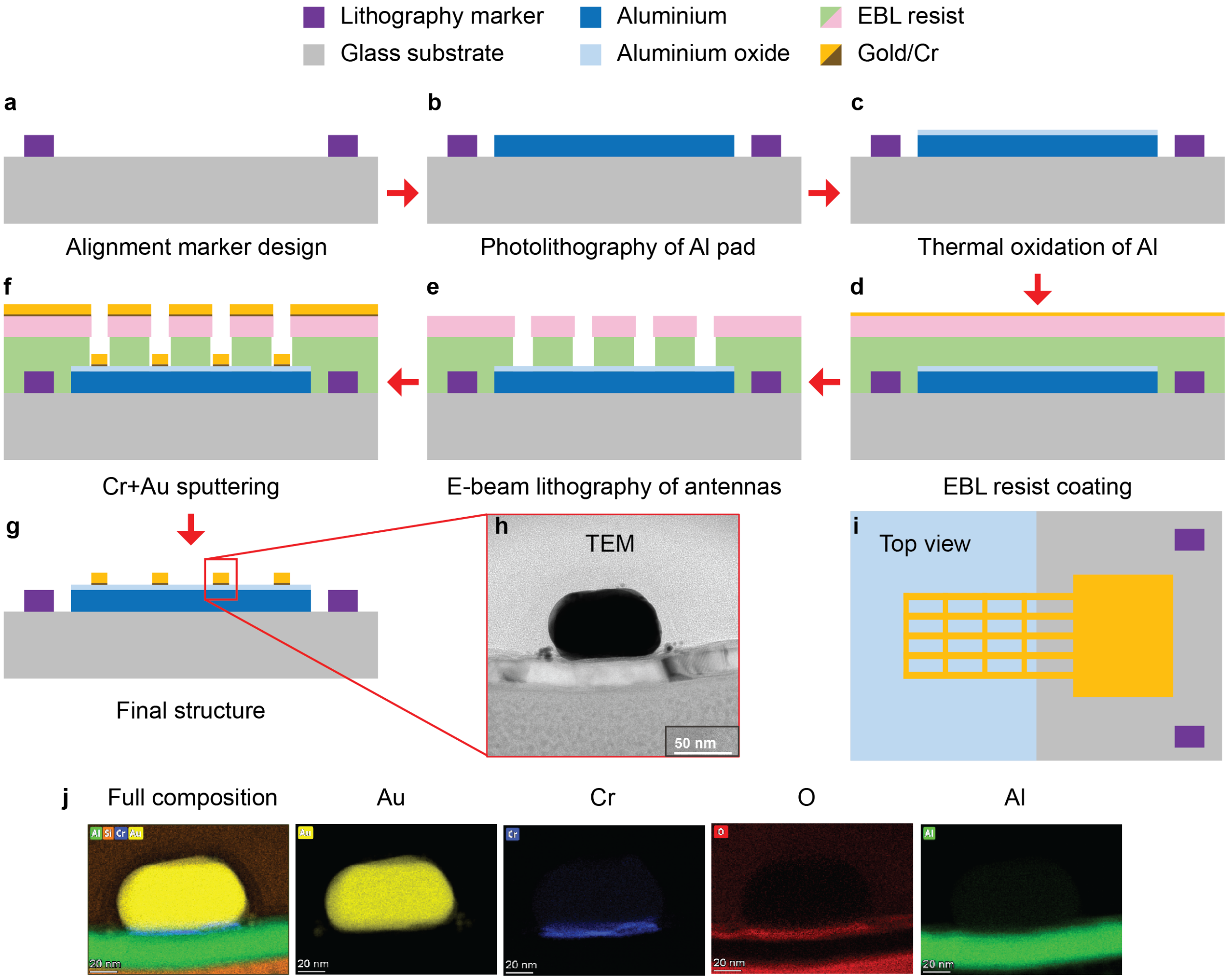

Figure S1: **Device fabrication.** **a-g**, Fabrication flow of self-illuminated plasmonic metasurfaces. **h**, Cross-sectional TEM image of a single nanowire antenna. **i**, Schematic top view of the device. **j**, EDS images of the antenna in (h), showing the spatial distribution of different elements.

and an atomic force microscope (AFM). As detailed in the main text and Fig. S2a, one of the target analytes (PMMA 495k A2) was patterned using e-beam lithography and lifted off using a developer solution (MiBK:IPA=1:3). Area 1 (green colored box) featured a mesh metasurface without analyte, while Area 2 (red colored box) had a mesh metasurface coated with analyte PMMA. Both of these areas were characterized by EMCCD to compare the intensity of the generated light through the LIET device, as illustrated in Fig. S2b. The mesh metasurface alone had a height of approximately 55 nm, whereas the analyte had a thickness of about 45 nm, as measured by AFM and depicted in Fig. S2c.

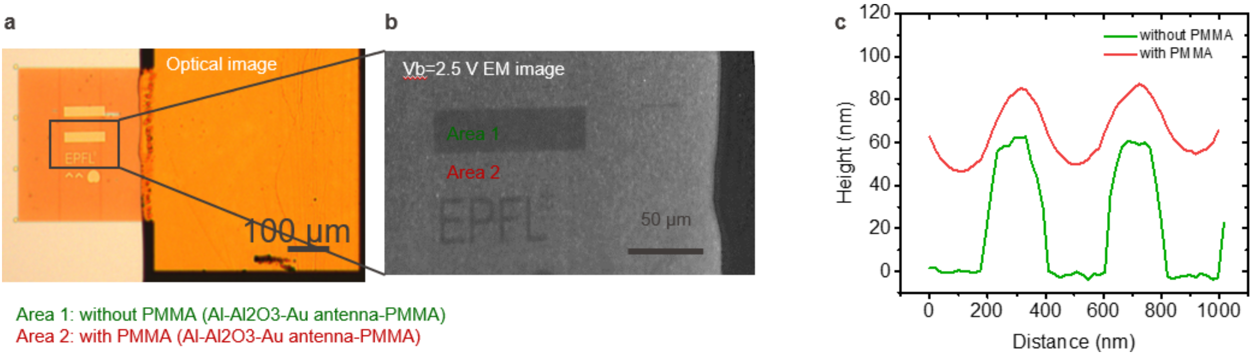

Figure S2: **Analyte characterization.** Characterization of surface features after functionalization of the LIET device with PMMA as measured by **a**, an optical microscope, **b**, EMCCD, and **c**, antenna line profiles with and without the analyte extracted from the 2D AFM scans.

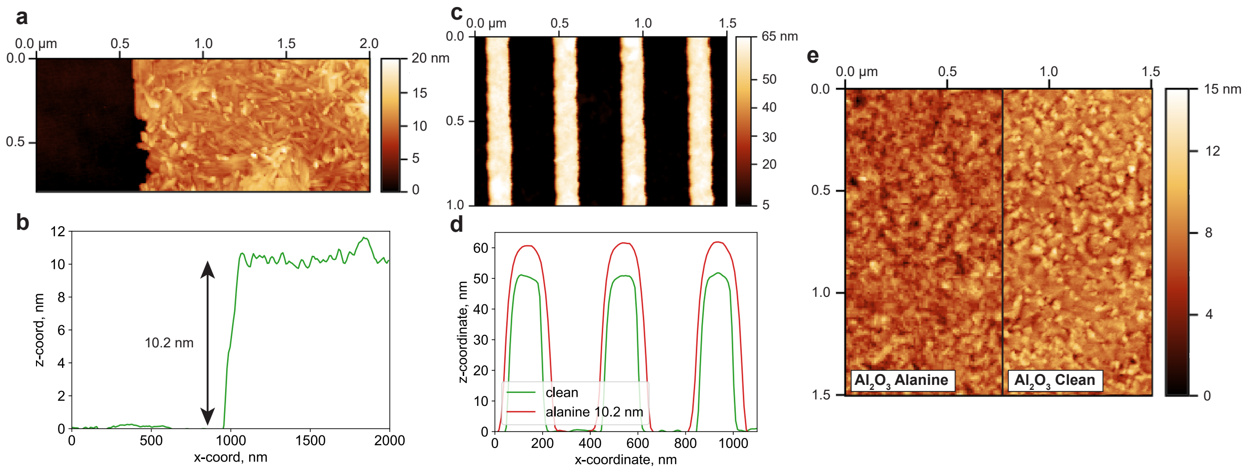

Figure S3: **AFM study of alanine evaporation on a LIET device and reference chips.** **a**, AFM scan image of the reference silicon chip containing bare and alanine coated regions. **b**, AFM line profile indicating the evaporated analyte thickness. **c**, AFM scan image of the pristine LIET device. **d**, AFM profiles of the LIET device before (green curve) and after (red curve) alanine evaporation. **e**, AFM scan images of the pristine Al_2O_3 surface (right) and after alanine evaporation (left).

As an analyte for biosensing, we used alanine, which is an α-amino acid with the chemical formula $C_3H_7NO_2$. Small amounts of alanine were deposited on the sample surface uniformly using thermal evaporation in a controllable manner. As shown in Fig. S3, we observed that alanine has different adhesion on different types of surfaces, which we confirmed with AFM measurements. Specifically, we found that, while the thickness of alanine films on the reference silicon chips (inserted together in the chamber with the LIET samples) and on

the surface of gold antennas is similar, there is no alanine adhesion on the alumina surface. This led to analyte deposition on the gold nanowire surface, thus closely emulating common biofunctionalization protocols (e.g., the ones based on thiol chemistry[1], which are selective for gold surfaces). Figure S3a is an AFM image taken on the silicon chip. The line profile in Fig. S3b indicates a uniform alanine coverage with ∼10.2 nm thickness. Figure S3c is an AFM image on the LIET device from the same evaporation session. The line profile shown in Fig. S3d corresponds well with the nominal nanowire height of 50 nm (from clean chip, green curve), while the data from the alanine-covered area shows an increase of the antenna height by an additional 10 nm, which matches well with the alanine thickness of 10.2 nm measured on the silicon reference. Finally, Fig. S3e shows the AFM images of alumina surfaces. The morphology of the surfaces for both pristine (right) and alanine-evaporated (left) chips are almost identical, indicating no alanine adhesion in the latter.

To correlate the sensitivity performance of our device with state-of-the-art methods, we used an optimized nanoplasmonic biosensor consisting of gold nanohole arrays (NHAs). The optimized NHA design has been successfully used in our previous biosensing experiments for thin biolayer detection[2]. We characterized its bulk sensitivity as 630 nm/RIU, which is well within the range reported in the literature[3], and thus, it is a good representative of a state-of-the-art. We covered the surfaces of both LIET and NHA biosensors with the same thickness of alanine (∼10.2 nm) in the same evaporation session and measured their spectral response with alanine in comparison to the clean samples (Fig. S4a and Fig. S4c). For LIET, we obtained the change in differential emission intensity within the selected spectral window of 620–700 nm (Fig. S4b). For NHA, we extracted the corresponding intensity change resulting from the analyte-induced redshifting of the extraordinary optical transmission (EOT) resonance peak (Fig. S4d). The results indicate a comparable amplitude of differential sensor response (∼0.2 for the LIET device *vs.* ∼0.6 for the NHA array as their peak values). Furthermore, if we consider the total amount of deposited analyte (which is ∼3 times more in the NHA due to the poor adhesion to alumina on the LIET device), the response am-

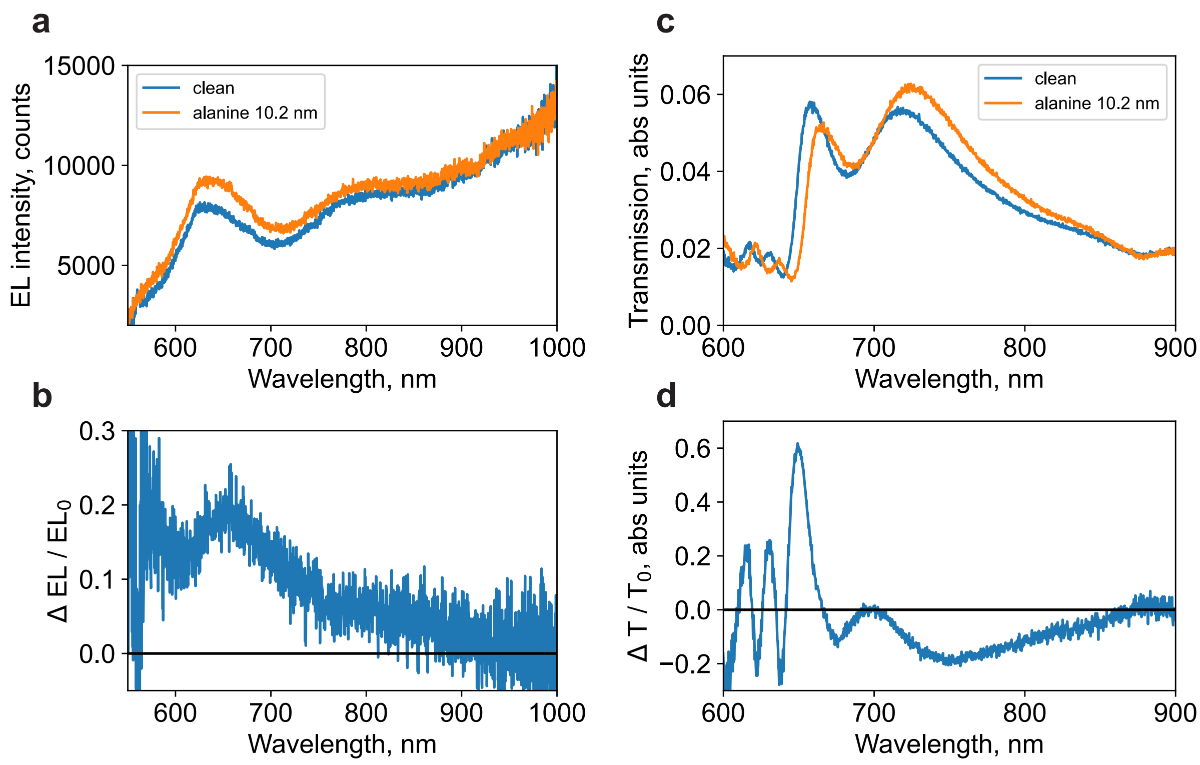

Figure S4: **Comparison of sensitivity performance of LIET device and a Au nanohole array (NHA) sensor. a**, Emission spectra of the pristine device (blue curve) and the same device after evaporation of a 10.2-nm thick alanine film (orange curve), corresponding to a total analyte mass of ∼18 pg on the measured device area. **b**, Spectral dependence of the differential LIET sensor response. **c**, Transmission spectra of the pristine Au nanohole array (blue curve) and the same device after alanine evaporation (orange curve). The total deposited mass of analyte on the NHA is estimated to be ∼54 pg. **d**, Differential transmission response of the Au nanohole array.

plitudes become almost identical. This highlights the potential of our LIET platform for high-performance biosensing.

S1.3 Electroluminescence characterization

We characterized the optical properties of each LIET device using a customized inverted optical microscope (Ti-E) equipped with a) an electron multiplying CCD (EMCCD, iXon Ultra 888) and b) a spectrometer (Isoplane 320 Spectrometer with a Pixis camera from Princeton Instrument). The optical characteristics were measured with 50× (NA=0.8) and

100× (NA=0.9) objectives when the junction was biased (i.e., Au biased and Al grounded). We employed a 300 EM gain to record EMCCD images for both the real and the back-focal planes. In Fig. S5a, the optical paths for the real plane (image plane) and back-focal plane images are indicated by green-solid and violet-solid lines, respectively. During the recording of back-focal plane images, an additional lens (Bertrand lens) was used in conjunction with the tube lens. To investigate polarization effects, an additional polarizer was introduced when recording both real and back-focal plane images.

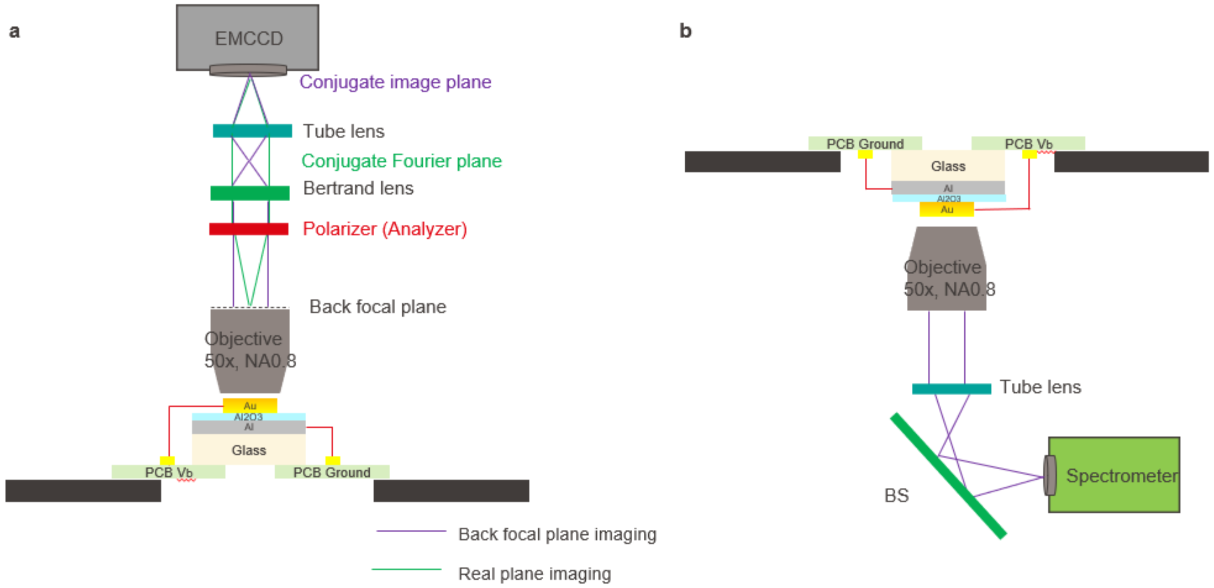

Figure S5: **Schematic of the electroluminescence characterization setup. a**, Optical path of real (green lines) and back-focal plane (violet lines) imaging. **b**, Optical path for spectrum measurement (violet lines) and device position relative to the objective.

For spectral measurements, the generated light was collected through the objective and then directed to the spectrometer, as shown in Fig. S5b. A correct interpretation of electroluminescence spectra necessitates taking into consideration the spectral transfer function of the detection path. The wavelength-dependent transfer function of all individual elements, as well as the quantum efficiency (QE) of the EMCCD, were obtained from the manufacturers. Using these data, corrected spectra and emitted photon numbers were calculated. This allowed us to obtain spectra that were independent of exposure time and pixel size,

corresponding to $\Delta\lambda$, and to form the final version of the spectrum, we derived corrected spectra according to

$$I_c = \frac{I_i - I_b}{I_t \Delta t \Delta \lambda}, \tag{S1}$$

where I_c, I_i, I_b, and I_t are the corrected, initial, background, and transfer-function intensities, respectively, while Δt represents the given exposure time, and each data point reflects the photon counts on individual pixels. The quantity $\Delta\lambda$ denotes the difference in nanometers between two consecutive measurement points. The units of the corrected intensity I_c are photon counts per second per nanometer of light wavelength. We remark again that this normalization procedure ensures that the spectrum is independent of exposure time and pixel size, providing a reliable measure of the emitted light intensity across different wavelengths. For additional optical characterization, an avalanche photodiode (APD, Excelitas Technologies SPCM-AQRH-14 APD) was employed to monitor photons emitted from the LIET devices under room temperature and ambient conditions. This APD setup was integrated into the laboratory of the ETH Zurich team, and its configuration was detailed in the antenna-coupled photon-emission experiment. All electrical excitation and characterization procedures were conducted using a Keithley 2636 B sourcemeter in conjunction with the Kickstart software from Linktronix. Both I-V (current-voltage) and I-t (current-time) modes were utilized, with current recording occurring typically every 100 ms. This comprehensive approach allowed for a thorough assessment of the electrical characteristics of the LIET devices.

S1.4 Stability of electrical and optical properties

The width and quality of the Al_2O_3 LIET device tunnel layer are expected to influence both the intensity and stability of the tunnel current. The field strength of our LIET devices was measured to be 4×10^9 V/m at room temperature under ambient conditions for a duration

of 13 minutes during which we maintained a voltage of 2.0 V. During the initial 10 minutes of recording, a minor increase in tunneling current was observed, with no significant fluctuations throughout the entire measurement period. Following the electrical stability test, the electroluminescence stability was assessed based on the applied voltage, comparing samples before and after the 13-minute electrical stability test. Despite the fact that experiments were conducted under ambient conditions, the tunnel junction demonstrated good stability in both electrical and optical characteristics, even with a relatively large LIET-based sensing area of approximately 300 μm $\times$ 300 μm.

S2 Electroluminescence modeling

S2.1 In-plane momentum conservation

In the derivation of the spectral electronic contribution

$$H(\omega, V_{\rm b}) \propto \int dE_{\rm f} \int dE_{\rm i}\, \rho^{\rm Al}_{E_{\rm i}}\, \rho^{\rm Cr}_{E_{\rm f}}\, f^{\rm Al}_{E_{\rm i}}\, (1 - f^{\rm Cr}_{E_{\rm f}})\, \delta\big[E_{\rm i} - (E_{\rm f} + \hbar\omega) + eV_{\rm b}\big] \tag{S2}$$

(Eq. (1) in the main text), we have imposed energy conservation during electron tunneling between the two metal gates. In this expression, e is the elementary charge, $V_{\rm b}$ is the bias voltage, ω is the emitted photon frequency, ρ^m_E is the electronic density of states of each metal m (Al and Cr) at an electron energy E, and f^m_E is the corresponding Fermi-Dirac distribution.

In addition to energy conservation, parallel momentum needs to be also conserved during the tunneling process. However, we anticipate that this condition may lead to minor corrections when dealing with polycrystalline surfaces that have sufficiently randomized grain orientations, although a tendency to expose surfaces with some preferred crystallographic orientation is generally expected in actual samples. As the films prepared in our experiments are polycrystalline, we present a calculation in which the average over surface orientations is

performed. To include momentum conservation in our model, Eq. (S2) needs to be modified to

$$H(\omega, V_\mathrm{b}) \propto \sum_{n_\mathrm{i},\mathbf{k}_\mathrm{i}} \sum_{n_\mathrm{f},\mathbf{k}_\mathrm{f}} \delta_{\mathbf{k}_{\mathrm{i}\parallel},\mathbf{k}_{\mathrm{f}\parallel}} \, f^{\mathrm{Al}}_{E_\mathrm{i}} \, (1 - f^{\mathrm{Cr}}_{E_\mathrm{f}}) \, \delta(E_\mathrm{i} - (E_\mathrm{f} + \hbar\omega) + eV_\mathrm{b}), \tag{S3}$$

where the integrals are replaced by discrete sums over electron wave vectors $\mathbf{k}_\mathrm{i}$ and $\mathbf{k}_\mathrm{f}$ for initial and final states in Al and Cr, respectively, and band indices n_i and n_f in each metal. Electron energies obviously depend on these indices (i.e., $E_\mathrm{i} \equiv E_{n_\mathrm{i}}(\mathbf{k}_\mathrm{i})$ and $E_\mathrm{f} \equiv E_{n_\mathrm{f}}(\mathbf{k}_\mathrm{f})$) according to the corresponding band structure (see details in Fig. S15). Momentum conservation is introduced through the Kronecker delta in Eq. (S3).

Since only the parallel component of the wave vector is conserved, the evaluation of Eq. (S3) requires prior knowledge of the crystalline orientation of both metal surfaces. Assuming random surface orientations in polycrystalline films, the average over such orientations permits transforming the Kronecker delta as

$$\delta_{\mathbf{k}_{\mathrm{i}\parallel},\mathbf{k}_{\mathrm{f}\parallel}} \quad \rightarrow \quad \int \frac{d\Omega_\mathrm{i}}{4\pi} \int \frac{d\Omega_\mathrm{f}}{4\pi} \, \delta_{\mathbf{k}_{\mathrm{i}\parallel},\mathbf{k}_{\mathrm{f}\parallel}} = \frac{1}{4\pi k_\mathrm{i} k_\mathrm{f}} \log\left(\frac{k_\mathrm{i} + k_\mathrm{f}}{|k_\mathrm{i} - k_\mathrm{f}|}\right). \tag{S4}$$

In Fig. S6, we compare the resulting spectral electronic contribution $H(\omega, V_\mathrm{b})$ with and without inclusion of in-plane momentum conservation for a bias voltage V_b in the 1.5–2.3 V range. Under positive bias, electrons tunnel from the polycrystalline Al layer to the polycrystalline Cr layer. As expected, the two results are nearly identical, so we conclude that parallel-momentum conservation can be disregarded for tunneling between polycrystalline metal surfaces, and only energy conservation needs to be incorporated.

S2.2 Calculation of the tunneling matrix elements

The electron tunneling characteristics presented in Fig. 3d of the main text are modeled considering tunneling between Al and Cr exclusively, thus not accounting for the presence of

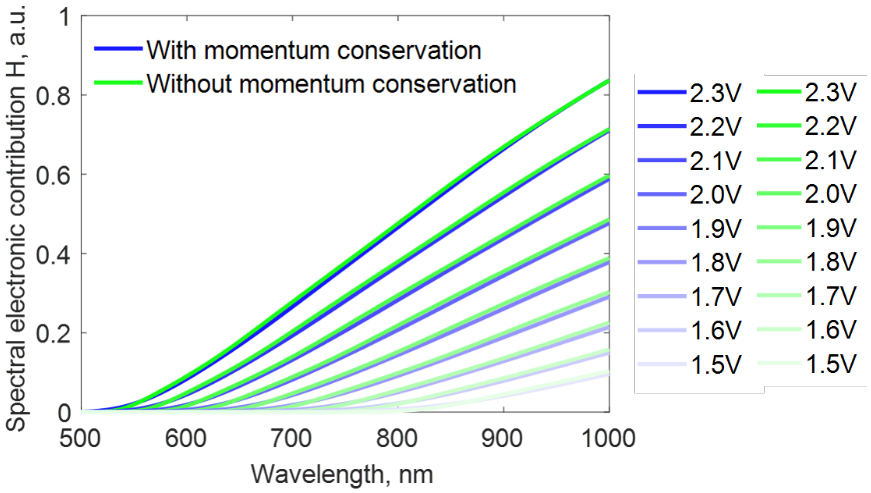

Figure S6: **Effect of in-plane momentum conservation.** Comparison of the spectral electronic contribution $H(\omega, V_{\rm b})$ calculated with and without imposing in-plane momentum conservation. Electrons tunnel from a polycrystalline Al layer to a polycrystalline Cr layer. The bias voltage $V_{\rm b}$ ranges from 1.5 V to 2.3 V.

Au. It is also possible that electrons from Al can tunnel into Au through the Cr layer, thereby contributing to the matrix elements describing the full tunneling process of our system, but a full account of the tunneling matrix elements incorporating such effects is complicated (it would require elaborate *ab initio* calculations of the interface averaged over a large number of crystal orientations and relative positions of the non-commensurate lattices of the two metals). To estimate the relative importance of both Au and Cr in the tunneling process, we calculate the corresponding functions $H_{\rm Al\rightarrow Cr}(\omega, V_{\rm b})$ for Al-to-Cr tunneling (same as in the main text) and $H_{\rm Al\rightarrow Au}(\omega, V_{\rm b})$ for Al to Au tunneling, and we employ an effective model in which the total spectral electronicic contribution is averaged as

$$H_{\rm ave}(\omega, V_{\rm b}) = H_{\rm Al\rightarrow Cr}(\omega, V_{\rm b})(1 - e^{-d/\lambda_{\rm e}}) + H_{\rm Al\rightarrow Au}(\omega, V_{\rm b})e^{-d/\lambda_{\rm e}}, \quad \text{(S5)}$$

where $d = 5$ nm is the thickness of the Cr layer and $\lambda_{\rm e}$ is the electron scattering mean free path in that material. To estimate the latter parameter, we take tabulated values[4] for the electric conductivity $\sigma = 7.9 \times 10^6$ S/m, the density $\rho = 7.19$ g/cm^3, and the atomic mass $A = 52$ g/mol of Cr. From here, assuming that this metal can be well-described

using the Drude model (and approximating the corresponding effective electron mass as the free-electron mass m_e), we calculate the electronic density of Cr as $n = \rho N_A/A = 8.33 \times 10^{22}$ cm^{-3} (N_A is the Avogadro's number) and subsequently the relaxation time of electrons in this metal as $\tau = m_e\sigma/ne^2 = 3.37$ fs. We also calculate the Fermi energy of Cr as $E_F = (\hbar^2/2m_e)(3\pi^2 n)^{3/2} = 6.95$ eV, from where the Fermi velocity follows as $v_F = \sqrt{2E_F/m_e} = 1.56 \times 10^6$ m/s (assuming parabolic bands). Finally, we are able to estimate the mean free path of electrons in Cr as $\lambda_e = v_F\tau = 5.26$ nm.

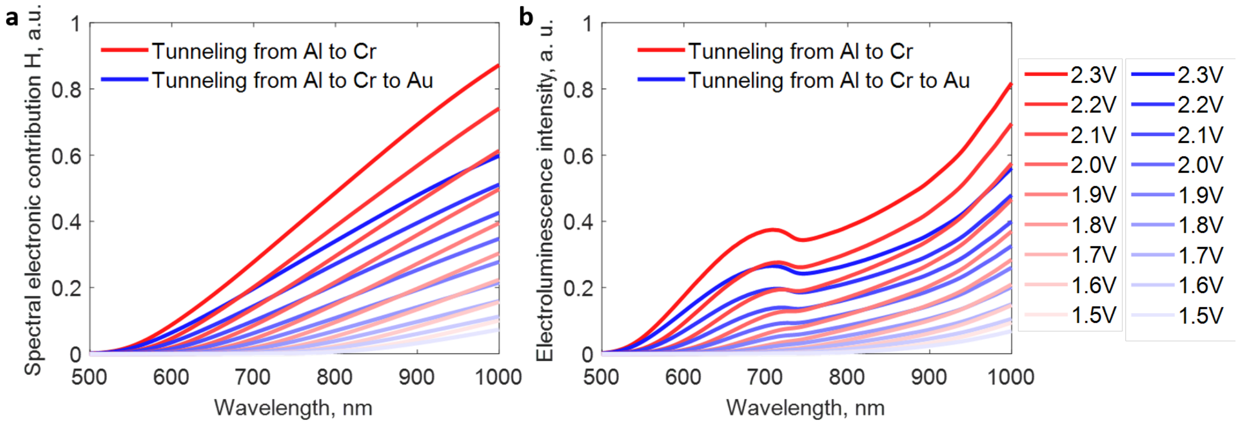

Figure S7: **The effect of the Au layer on electron tunneling a**, spectral electronic contribution and **b**, electroluminescence intensity comparing electron tunneling between Al and Cr, and tunneling from Al to Cr with subsequent propagation to Au. Simulations are performed for applied voltages ranging from 1.5 V to 2.3 V.

In Fig. S7, we show the results of such analysis by comparing the spectral electronic contribution and the electroluminescence signal computed using the effective model (H_{ave}, red curves) and the model employed in the main text ignoring the presence of Au ($H_{Al\to Au}$, blue curves), across a voltage range of 1.5 V to 2.3 V. We observe no significant spectral shift between the two calculations, and the differences in signal intensity are relatively small, indicating a minimal variation between the two configurations. We conclude that the approximation of neglecting the Au characteristics in evaluating the tunneling matrix elements is valid due to the large thickness of the Cr layer, which exceeds the mean free path of electrons therein.

S2.3 Optical modes supported by the system

The antenna mesh design proposed in the main text supports two dominant types of optical modes (namely, plasmonic antenna lattice modes and metal-insulator-metal gap modes) that contribute to the observed electroluminescence signal. To illustrate their influence on the observed electroluminescence spectra and the sensing performance, we carry out an in-depth numerical analysis of our structure.

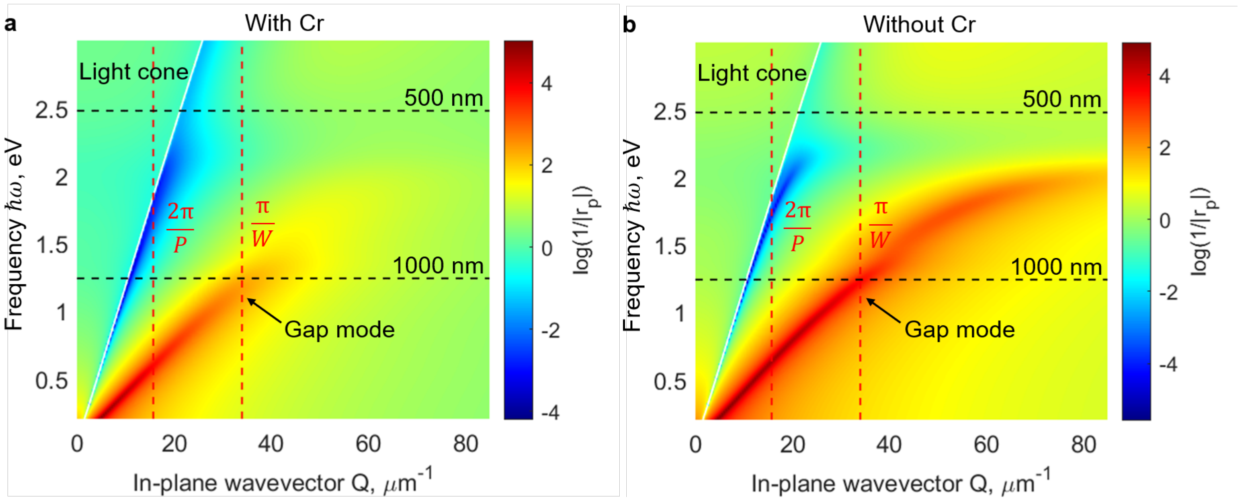

Figure S8: **Gap mode contribution analysis. a**, Dispersion relation of the gap mode calculated for a 1D structure consisting of infinite layers of Au (50 nm), Cr (5 nm), Al_2O_3 (5 nm), and Al (25 nm) following the material layer sequence from the main text. **b**, Same calculation as in **a** without the Cr Layer. Dashed vertical lines in both panels illustrate the bands defined by the period of the plasmonic lattice P (400 nm) and the width of the nanoantenna W (92 nm).

First, we calculate the dispersion relation of an infinitely extended Al/Al_2O_3/Cr/Au heterostructure (for the layer thicknesses reported in the main text). The results shown in Fig. S8a indicate that the gap mode supported by our system manifests mostly in the near-IR region (>1000 nm). Fig. S8b reveals that the presence of the Cr layer is responsible for the suppression of the gap mode at shorter wavelengths.

It is important to mention that, while the Cr layer suppresses the gap mode at shorter wavelengths, it also has a positive effect on the total electroluminescence intensity according to our simulations comparing the electroluminescence spectra of Al/Au and Al/Cr/Au tun-

neling junctions (Fig. S9). Simulations of the spectral photonic contribution G for Al/Au junction (Fig. S9a, blue curve) show a peak around 700 nm that is strongly quenched after the addition of a Cr layer (Fig. S9a, red curve). However, this is overcompensated by a much higher spectral electronic contribution (represented by the H function) of the Al/Cr/Au junction (Fig. S9b) due to a better matching of the band structures between the two materials. This leads to a higher total electroluminescence signal for the Al/Cr/Au junction (Fig. S9c).

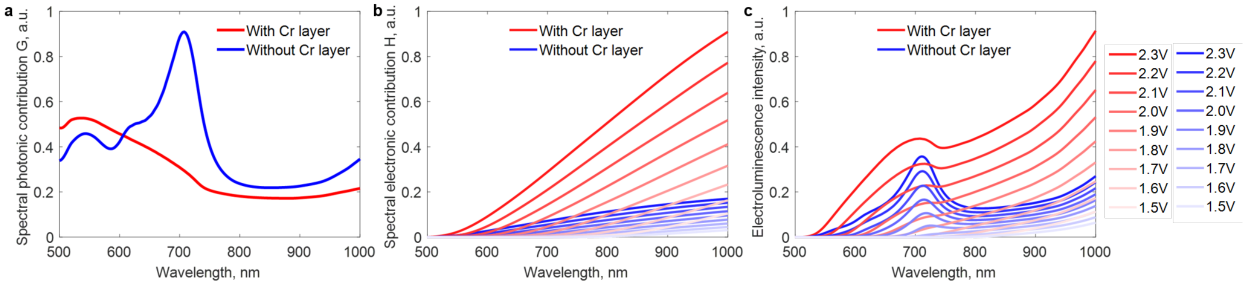

Figure S9: **Influence of the Cr layer on the total electroluminescence intensity.** Comparison of **a**, the spectral photonic contribution G for a signal collection NA of 0.8, **b**, the spectral electronic contribution H, and **c**, luminescence spectra for Al/Au (labeled: without Cr layer) and Al/Cr/Au (labeled with Cr layer) junctions. The structure with the Cr layer features a lattice with a 400 nm periodicity and a nanoantenna width of 92.5 nm, along with layer thicknesses of 50 nm for Au, 5 nm for Cr, 5 nm for Al_2O_3, and 25 nm for Al, all deposited on a glass substrate.

To elaborate on the contribution of the plasmonic lattice mode to the observed electroluminescence spectra, we perform numerical simulations of angle-resolved absorption spectra that are directly related to emission through the reciprocity principle. Figure S10a shows absorption maps calculated for different periods of the structure, plotted in $(k_0, k_{||})$ space. The lattice mode manifests as absorption maxima with a dependence on the angle of incidence and on the period of the structure, as indicated with white arrows in every frame of Fig. S10a. In the experiment, the electroluminescence signal is collected through an objective lens, which combines the contributions of emission from multiple angles and smears out the spectral features of the lattice mode. This is illustrated in Fig. S10b, which shows the absorption spectra of the structure for a discrete set of angles within the numerical aperture NA=0.8 of the objective used in the measurements. Each of these spectra at discrete angles

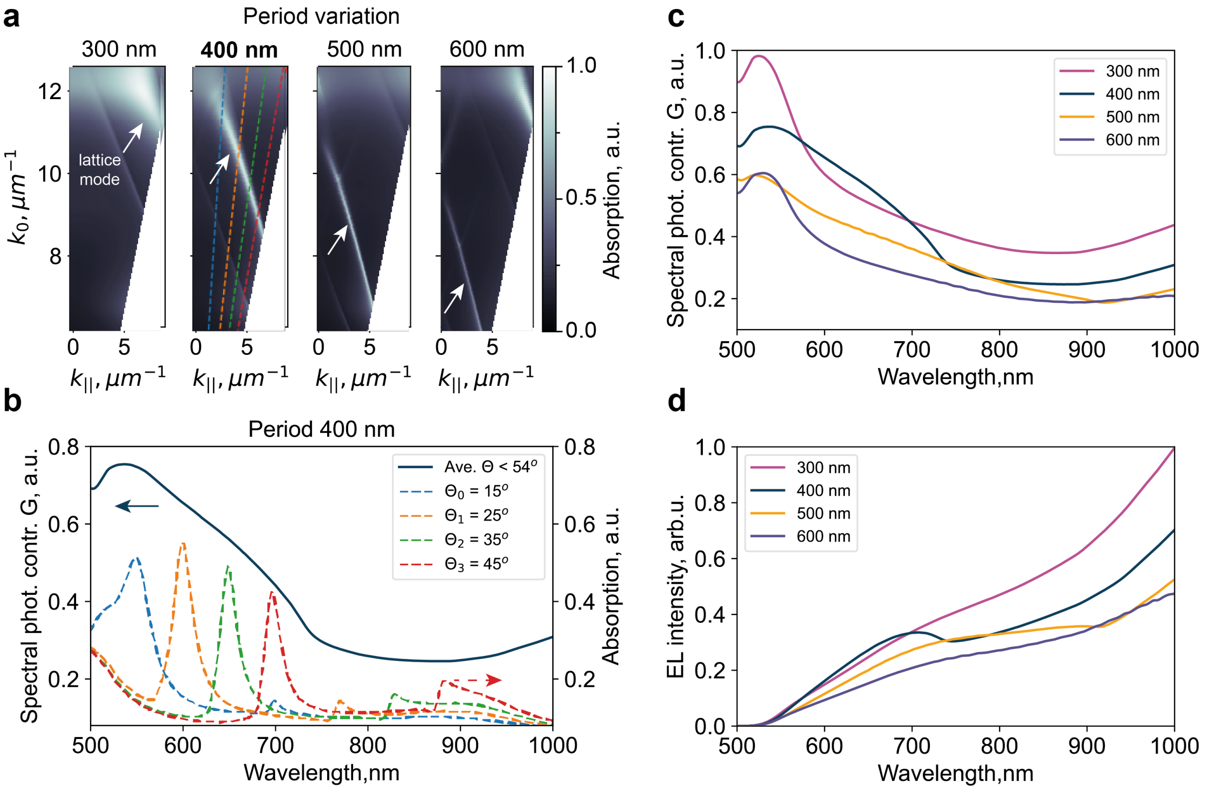

Figure S10: **Analysis of the contribution of the plasmonic lattice mode. a,** Angle-resolved absorption spectra of the plasmonic nanoantenna array calculated with the Fourier modal method for different periods of the lattice. The antenna width is fixed at 92.5 nm and the gap thickness at 5 nm. The dispersive plasmonic lattice mode is marked with arrows in each panel. **b**, Dashed curves: calculated absorption spectra of the plasmonic lattice with 400 nm for discrete angles of incidence (also marked with dashed lines in panel **a**). Solid curve: spectral photonic contribution averaged over the full numerical aperture covered by the objective used in the experiment (NA=0.8, $\theta_{\max} = 54°$). **c**, Spectral photonic contribution $G(\omega)$ for NA=0.8 calculated for different periods of the plasmonic nanoantenna lattice. **d**, Total electroluminescence intensity calculated for a bias voltage of 2.3 V under the conditions of panel a.

(dashed curves) has a sharp peak corresponding to the lattice mode, and when averaged over the full NA, it transforms into a broader spectral feature that we observe in the $G(\omega)$ spectra (solid curve). This also leads to a less prominent dependence of $G(\omega)$ and the total electroluminescence intensity on the lattice period, as illustrated in Fig. S10c,d.

The simulations of the electroluminescence intensity over a broader spectral range shown in Fig. S11. indicate that the gap mode is associated with the emission intensity increase at longer wavelengths. The spectra show a prominent long wavelength peak that depends

on both the nanowire width (Fig. S11a) and the gap thickness (Fig. S11b) that define the parameters of the Fabry-Pérot cavity for the gap plasmon[5].

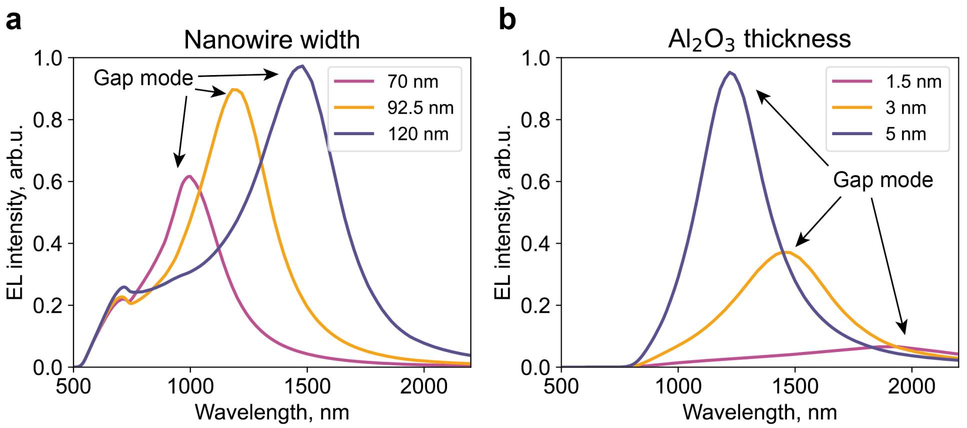

Figure S11: **Analysis of the contribution of the gap mode.** Spectra of electroluminescence for the plasmonic nanoantenna array with 400 nm period biased at 2.3 V and calculated for **a**, different antenna widths at a constant gap thickness of 5 nm and **b**, different gap thicknesses at a constant antenna width of 92.5 nm.

S2.4 Electromagnetic mode analysis through the angle-resolved emission

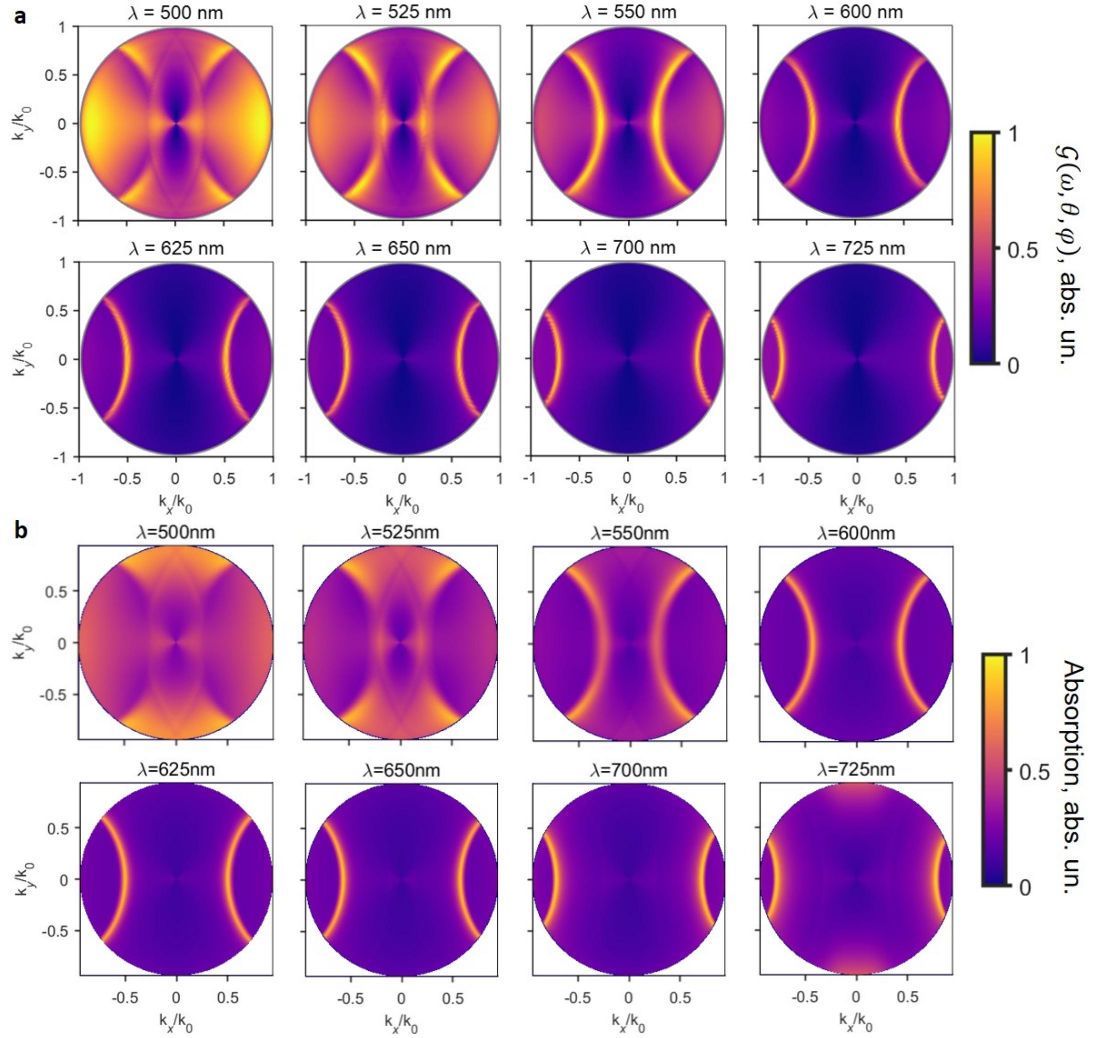

Figure S12: **Momentum-space maps.** **a**, Angle-resolved spectral photonic contribution $\mathcal{G}(\omega, \theta, \phi)$ of a LIET device and **b**, (k_x, k_y) maps of absorption for plane-wave excitation calculated with the Fourier modal method for selected wavelengths ranging from 500 nm to 725 nm. The angle-resolved spectral photonic contribution is normalized to the maximum in each map. We show results up to a maximum polar angle $\theta = 54°$, awhich corresponds to an objective with a numerical aperture NA = 0.8. The nanoantenna structure has a periodicity of 400 nm, a ribbon width of 92.5 nm, and layer thicknesses of 50 nm Au, 5 nm Cr, 5 nm Al_2O_3, and 25 nm Al on top of a glass substrate.

To analyze the angular distribution of emission, we study the full angular patterns of the spectral photonic contribution $\mathcal{G}(\omega, \theta, \phi)$ at selected wavelengths ranging from 500 nm

to 725 nm. The function $\mathcal{G}(\omega, \theta, \phi)$, which is defined in Eq. (3) in the main text, is given by

$$\mathcal{G}(\omega, \theta, \phi) = \sum_l \left| \sum_j g(\hat{\mathbf{r}}, \mathbf{r}_{lj}, \omega) \right|^2 \tag{S6}$$

We remind that the excitation of a plasmonic mode by light incident from a given direction is connected by the reciprocity principle to the emission of light along that direction via radiative de-excitation of the same mode, which is considered to be previously excited by inelastic electron tunneling in the present context. Due to these reciprocity considerations, the full-wave numerical simulations of the $\mathcal{G}$ function performed in COMSOL (Fig.S12a) show excellent agreement with the angle-resolved absorption patterns calculated with the Fourier modal method (Fig. S12b). The polar emission maps reveal the isofrequency contours of the dispersive lattice mode, which shift to larger emission angles with increasing wavelength, consistent with Fig. S10a.

To further investigate the contribution of the plasmonic lattice mode to the emission and sensing mechanisms of our devices, we experimentally measure the angle-resolved emission spectra using a back-focal plane spectroscopy setup and corroborate the data with numerical simulations. Figure S13 illustrates the modifications of the measured emission spectra due to the presence of the analyte. The left-half part of Fig. S13a and Fig. S13d shows the angle-resolved emission from a clean sensor. After the deposition of a thin alanine film with an equivalent deposited mass of 8.5 pg, which is close to the detection limit of our sensor, the emission spectra are modified as shown in the right-half part of Fig. S13a and Fig. S13d. To highlight this change, we plot the difference of the measured and modeled emission intensity with and without the analyte in Fig. S13b and Fig. S13e respectively, which reveals that the analyte induces a spectral redshift of the lattice mode (decrease of k_0 in the axes used in Figs. S13b and S13e). This is further illustrated in the emission spectra extracted for a discrete set of angles indicated with light colored lines in Fig. S13a and Fig. S13d. These spectra, shown in Fig. S13c and Fig. S13f respectively, indicate that

distinct peaks (cf. angle-resolved absorption spectra in Fig. S10b) experience around 10 nm spectral shift (dark colored curves). When the emission is collected within the full NA of the objective, this redshift leads mainly an increase in emission intensity, as shown in Fig. 4e,f of the main text.

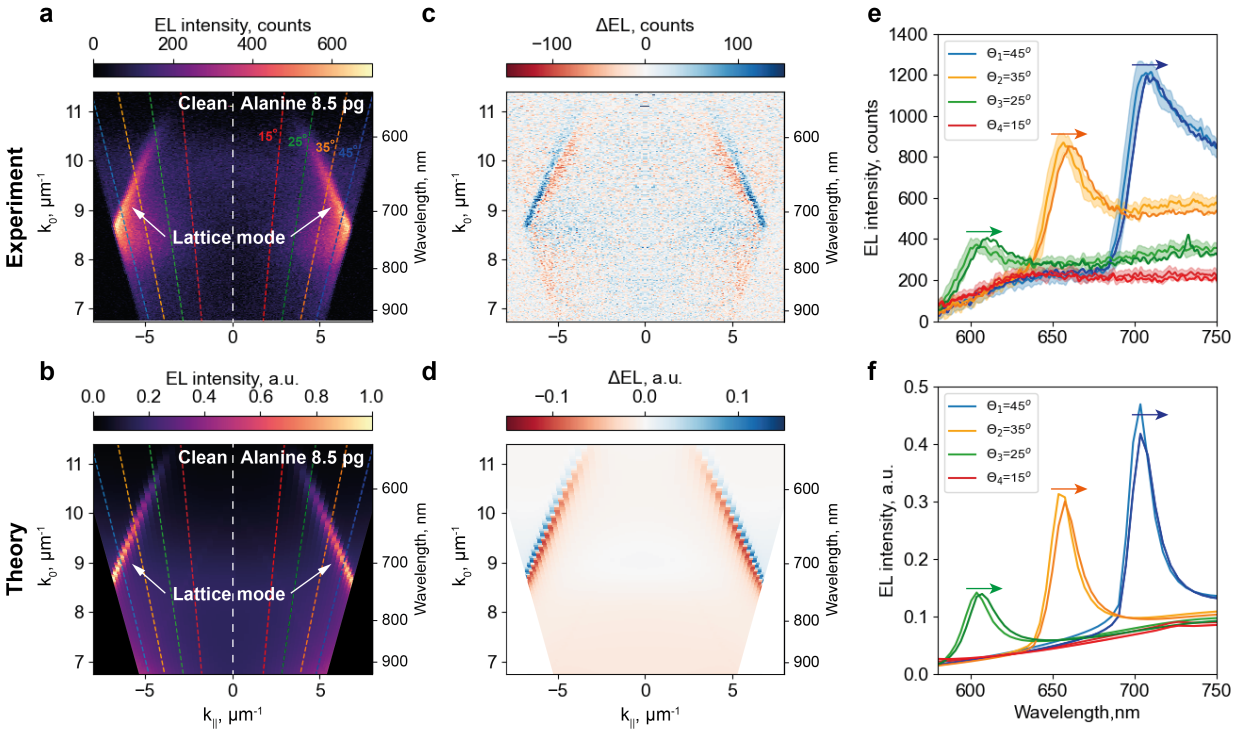

Figure S13: **Detection of analyte in angle-resolved emission spectra.** **a**, Measured and **b**, numerically calculated angle-resolved emission from a clean sensor (left half) and the same sensor covered with a thin alanine film (equivalent mass of 8.5 pg, right half). The angle-dependent emission peaks originate from the photonic lattice mode (compare with Fig. S10a). **c,d** Measured and simulated differential angle-resolved emission maps with and without the analyte, highlighting the spectral redshift of the emission peaks induced by the analyte. **e,f** Comparison of the emission spectra at a discrete set of angles in experiment and simulation. The data for a clean sensor are shown with light colored curves, while the data with 8.5 pg of alanine are shown with dark colored curves. The respective angles are marked with dashed lines in panels a,b.

S2.5 Contribution of the leakage radiation channel

To estimate the total electron-to-photon conversion efficiency of our device taking into account all radiative channels (in particular, the leakage radiation channel), we calculate the spectral photonic contribution $G(\omega)$ for all angles covering both the upper (emission into the air) and the lower (emission into the substrate) half-spaces. The results are presented in Fig. S14a, with the spectral photonic contribution computed separately for a collection objective with NA=0.8 ($\theta_{max} = 54°$) as well as for the full upper and lower half-spaces. The normalized data in Fig. S14b shows that we collect an average of 35% of the radiation in our experiments. At the same time, the contribution of the leakage radiation channel on average does not exceed 15% of the total emission of the structure. With these data, we estimated the total electron-to-photon conversion efficiency of our device (1.2×10^{-7} photons per electron) by taking into account all radiative channels.

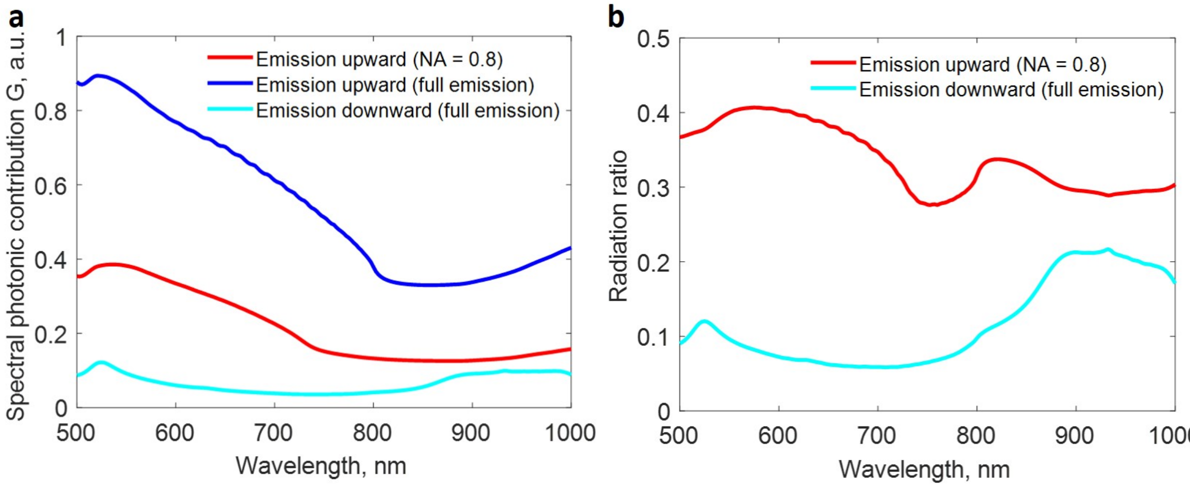

Figure S14: **a**, Calculated spectral photonic contribution of the metasurface with 92.5 nm wide antennas for collection from the top with NA=0.8 (red curve) compared with the full emission in the upper (blue curve) and lower (light blue curve) half-spaces. **b**, Calculated emission in the upper half-space with NA=0.8 (red curve) and emission into the substrate (light blue curve), both normalized to the total emission from the structure.

S2.6 Electronic band structure and density of states

The electronic density of states and band structure of Al[6] and Cr[7] (entering the evaluation of Eqs. (S2) and (S3)) are reproduced in Fig. S15, as extracted from a repository of density-functional-theory (DFT) calculations.[8,9] The counterpart functions for Au, necessary for the evaluation of Eq. (S5), are retrieved from the same source[10]. We also represent the distributions $f^{\rm Al}_{E_{\rm i}}$, $1 - f^{\rm Cr}_{E_{\rm f}}$, and $f^{\rm Al}_{E_{\rm i}}(1 - f^{\rm Cr}_{E_{\rm f}})$ derived from the Fermi-Dirac distributions for Al and Cr, assuming energy conservation according to Eq. (S2) (i.e., $E_{\rm i} - (E_{\rm f} + \hbar\omega) + eV_{\rm b}$) for a bias voltage $V_{\rm b} = 2.3$ V and a light wavelength of 650 nm (Fig. S15b).

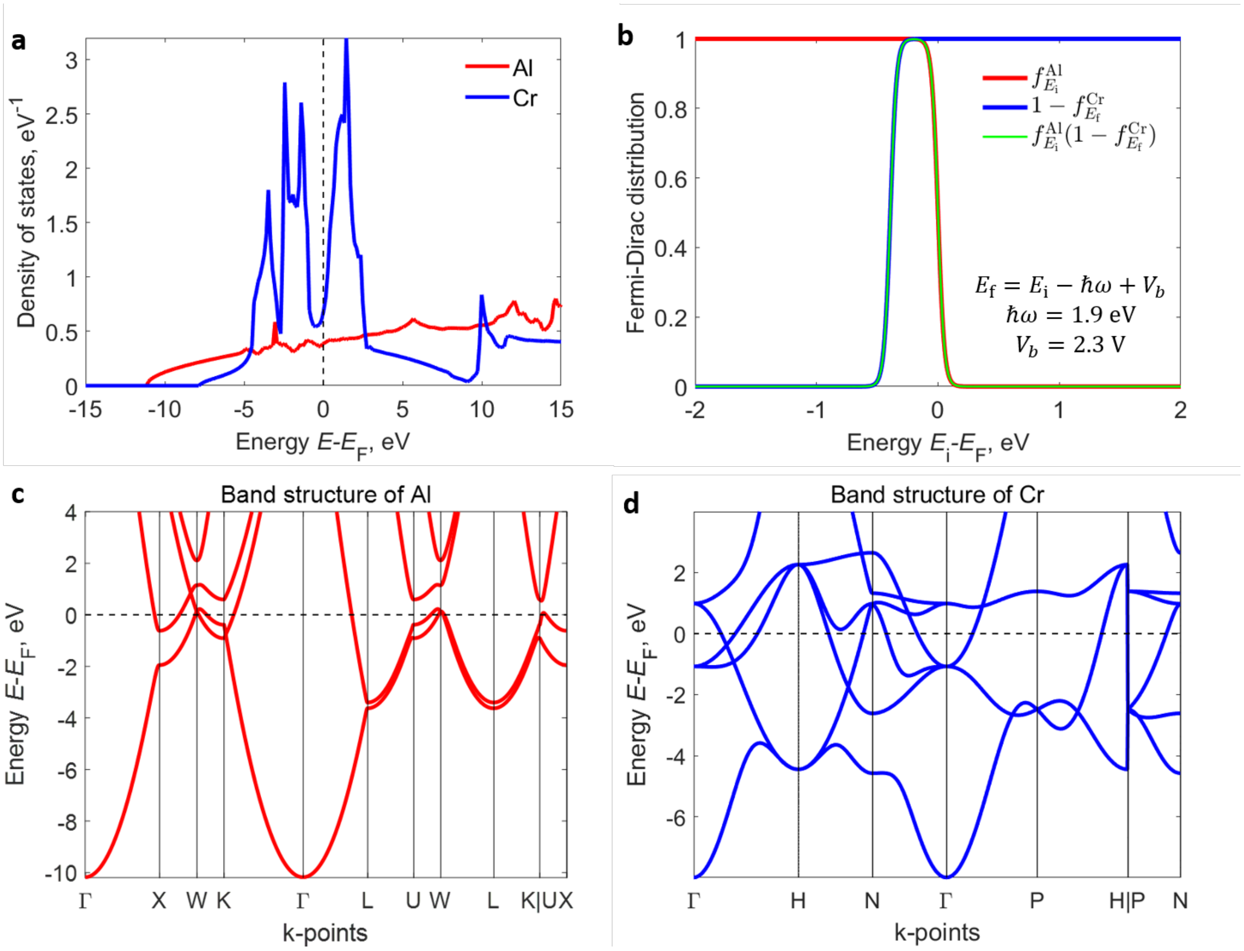

Figure S15: **Electronic density of states, band structures, and Fermi-Dirac distributions for Al and Cr. a**, Electronic density of states of Al and Cr, with the energy referred to the Fermi energy $E_{\rm F}$ in each of these metals. **b**, Fermi-Dirac distributions $f^{\rm Al}_{E_{\rm i}}$, $1 - f^{\rm Cr}_{E_{\rm f}}$, and $f^{\rm Al}_{E_{\rm i}}(1 - f^{\rm Cr}_{E_{\rm f}})$ (see Eq. (S2)) at a wavelength of 650 nm for a bias voltage $V_{\rm b} = 2.3$ V. **c,d**, Electronic band structures of Al (c) and Cr (d).

References

(1) John-Herpin, A.; Kavungal, D.; von Mücke, L.; Altug, H. Infrared Metasurface Augmented by Deep Learning for Monitoring Dynamics between All Major Classes of Biomolecules. *Adv. Mater.* **2021**, *33*, 2006054.

(2) Li, X.; Soler, M.; Szydzik, C.; Khoshmanesh, K.; Schmidt, J.; Coukos, G.; Mitchell, A.; Altug, H. Label-Free Optofluidic Nanobiosensor Enables Real-Time Analysis of Single-Cell Cytokine Secretion. *Small* **2018**, *14*, 1800698.

(3) Altug, H.; Oh, S.-H.; Maier, S. A.; Homola, J. Advances and applications of nanophotonic biosensors. *Nat. Nanotechnol.* **2022**, *17*, 5–16.

(4) Kenneth Barbalace. Periodic Table of Elements. `https://EnvironmentalChemistry.com/yogi/periodic/Cr.html`, Accessed: 2024-10-21.

(5) Zhang, C.; Hugonin, J.-P.; Coutrot, A.-L.; Sauvan, C.; Marquier, F.; Greffet, J.-J. Antenna surface plasmon emission by inelastic tunneling. *Nat. Commun.* **2019**, *10*, 1–7.

(6) The Materials Project, Materials Data on Al by Materials Project. **2020**,

(7) The Materials Project, Materials Data on Cr by Materials Project. **2020**,

(8) Jain, A.; Ong, S. P.; Hautier, G.; Chen, W.; Richards, W. D.; Dacek, S.; Cholia, S.; Gunter, D.; Skinner, D.; Ceder, G.; Persson, K. A. Commentary: The Materials Project: A materials genome approach to accelerating materials innovation. *APL Materials* **2013**, *1*, 011002.

(9) Munro, J. M.; Latimer, K.; Horton, M. K.; Dwaraknath, S.; Persson, K. A. An improved symmetry-based approach to reciprocal space path selection in band structure calculations. *npj Computational Materials* **2020**, *6*, 112.

(10) The Materials Project, Materials Data on Au by Materials Project. **2020**,
